# Liquid Biopsy: Past, Present, and Future

**DOI:** 10.1002/mco2.71085

**Published:** 2026-10-27

**Authors:** Jiachen Liu, Dianjie Zeng, Keran Zhao, Yuxi Wang, Huijie Hu, Fei Deng, Yinhuai Wang, Liya Sun, Liangmin Fu

**Affiliations:** ^1^ Department of Urology The Second Xiangya Hospital at Central South University Changsha Hunan China; ^2^ Xiangya School of Medicine Central South University Changsha Hunan China; ^3^ Department of Urology Xiangya Hospital Central South University Changsha Hunan China; ^4^ Washington University School of Medicine St Louis Missouri USA; ^5^ Guangdong Medical University Dongguan Guangdong China; ^6^ Department of Nephrology Xiangya Hospital at Central South University Changsha Hunan China; ^7^ National Clinical Research Center for Metabolic Disease Key Laboratory of Diabetes Immunology (Central South University) Ministry of Education Changsha Hunan China

**Keywords:** circulating tumor cells (CTCs), circulating tumor DNA (ctDNA), extracellular vesicles (EVs), liquid biopsy, minimal residual disease (MRD), multicancer early detection (MCED)

## Abstract

Liquid biopsy has evolved over two decades from an extension of tissue genotyping into a minimally invasive approach for repeat and longitudinal molecular assessment across the cancer continuum. Advances in sequencing, digital PCR, error suppression, and computational analytics have expanded analytes beyond circulating tumor DNA and circulating tumor cells to extracellular vesicles, circulating RNAs, and tumor‐educated platelets, while broadening readouts from mutations to methylation, fragmentomics, transcriptomics, proteomics, and multiomic integration. This review traces the field from its technological foundations to current applications in early detection and multicancer early detection, therapy selection and resistance monitoring, minimal residual disease, recurrence‐risk stratification, and longitudinal response assessment across major cancers and selected nononcologic settings. We distinguish clinically established applications from emerging and exploratory strategies and emphasize that high analytical performance does not by itself establish clinical utility. We further discuss preanalytical handling, standardization, biological confounders, and evidence required for implementation. Future progress will depend on use‐case‐driven development, prospective utility studies, longitudinal and multimodal interpretation, and integration with established diagnostic pathways. Together, these priorities define a transition from molecular detectability toward reproducible, actionable, and clinically meaningful liquid biopsy.

## Graphical abstract

Liquid biopsy has expanded from cancer genotyping to a broader strategy for probing both oncologic and nononcologic conditions through circulating analytes and multilayer molecular readouts. This review traces the field's past, present, and future—summarizing where liquid biopsy is most actionable today, why performance varies across contexts, and which technical and implementation advances are needed for robust, standardized clinical use

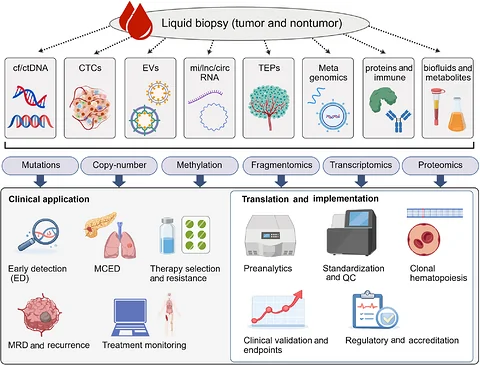

## 1 Introduction

Liquid biopsy has undergone a pivotal transition over the past two decades, evolving from a conceptual idea into a clinical tool. By extending tumor‐related information from a “local tissue snapshot” to a “system‐wide, dynamic window,” it enables serial, minimally invasive, and repeatable molecular measurements [1]. The early rapid expansion of the field was propelled by the discovery of key analytes—most notably circulating tumor DNA (ctDNA) and circulating tumor cells (CTCs)—together with exponential advances in detection technologies [2]. More recently, additional biological carriers, including exosomes/extracellular vesicles (EVs), circulating RNA, and tumor‐educated platelets (TEPs) [3, 4, 5, 6], have come into focus, further shifting liquid biopsy from a “mutation readout” toward a multidimensional “tumor ecosystem readout,” integrating information across epigenetic profiling, fragmentomics, transcriptomics, and proteomics. With the maturation of sequencing, digital PCR, error‐suppression strategies, and computational bioinformatics, liquid biopsy has steadily moved from a research instrument to multiple critical nodes along the clinical pathway.

Yet, the “present” landscape of liquid biopsy remains both vibrant and highly heterogeneous. Different analytes vary substantially in the types of information they carry, their spatiotemporal representativeness, and their technical vulnerabilities; even for the same clinical question, signal strength and noise structure differ across cancer types, disease stages, and treatment contexts [7, 8]. Accordingly, the most pressing clinical questions have shifted from “can we detect it?” to “is what we detect sufficient to change decisions?” In early detection and multicancer early detection (MCED), the challenge is to jointly optimize sensitivity and specificity under conditions of low tumor burden and high background noise, while addressing false positives, overdiagnosis, and uncertainty in downstream follow‐up pathways [9, 10]. In therapy selection and resistance monitoring, the task is to establish actionable causal links between molecular changes and treatment response, while avoiding misclassification driven by platform variability and confounders such as clonal hematopoiesis [11]. In minimal residual disease (MRD) assessment and recurrence prediction, detection thresholds and sampling time points must be aligned with clinically meaningful endpoints to enable strategies that are verifiable, reimbursable, and scalable [12]. Meanwhile, “invisible engineering details”—including preanalytical variables, sample handling workflows, quality control, and regulatory compliance—often determine reproducibility and reliability more directly than new algorithms or novel biomarkers [13].

Looking ahead, the central trajectory of liquid biopsy is unlikely to be the replacement of one analyte by another, but rather complementary integration around specific clinical tasks: selecting the most appropriate information carriers and measurement dimensions for the question at hand, and translating research signals into implementable clinical workflows through coherent evidence grading and standardization. Guided by this perspective, this review follows a “past–present–future” narrative to provide a structured synthesis of the evolution of key liquid biopsy analytes and methodologies; summarize the clinical evidence, limitations, and evidence maturity across major tumor types and selected nononcologic settings; and highlight the practical determinants of real‐world implementation, including preanalytical and quality systems, regulatory considerations, and translational pathways (Figure 1). This article is a clinically oriented narrative review based on a targeted literature search, with emphasis on landmark studies, clinical validation studies, professional guidelines, regulatory documents, and recent high‐quality evidence. Rather than providing an exhaustive catalogue of reported biomarkers, we aim to distinguish clinically established applications from emerging approaches and to clarify the evidence required for further translation.

**FIGURE 1:**
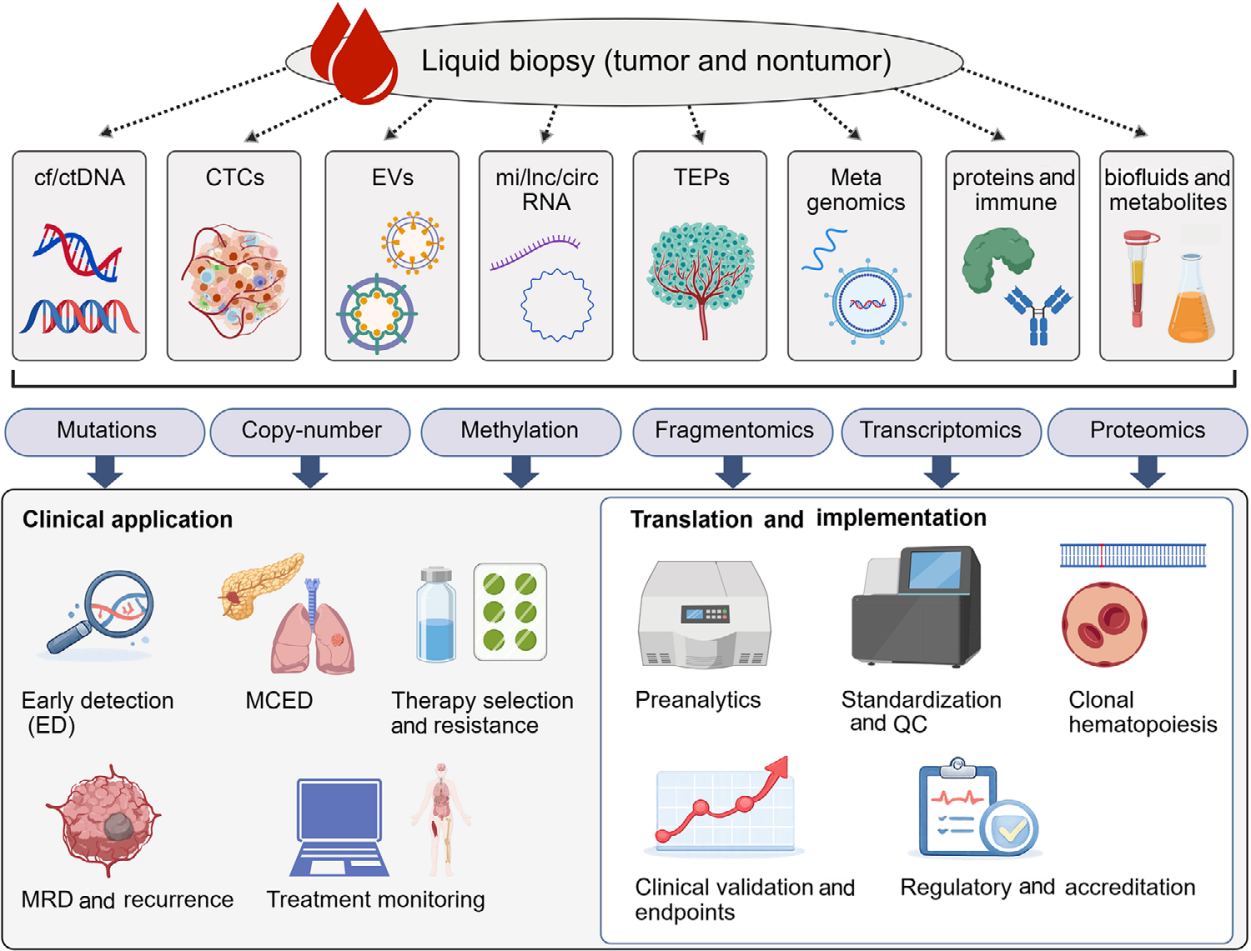
Conceptual framework linking liquid‐biopsy analytes to clinical applications and translational requirements. The framework summarizes major circulating analytes, including cfDNA/ctDNA, CTCs, extracellular vesicles, circulating RNAs, tumor‐educated platelets, and selected nononcologic cfDNA signals, together with their principal molecular readouts and clinical use cases. Translation into practice depends on preanalytical standardization, analytical and clinical validation, demonstration of clinical utility, and integration with established diagnostic pathways.

## 2 Historical Evolution and Methodological Advances of Liquid Biopsy

Liquid biopsy has evolved from the initial observation of circulating nucleic acids to a clinically deployable discipline enabled by advances in molecular biology, sequencing, microfluidics, and computational error suppression. Conceptually, this evolution can be viewed as a sequence of “biological layers” added over time: (i) acellular nucleic acids (cell‐free DNA [cfDNA]/ctDNA), (ii) intact cellular analytes (CTCs), and (iii) vesicular and host‐response signals (EVs, noncoding RNAs [ncRNAs], and TEPs) (Figure 2). This layered expansion reflects a central theme in the field: no single analyte is universally optimal, and each new class of biomarker emerged to address limitations in sensitivity, specificity, or biological interpretability of earlier approaches.

**FIGURE 2:**
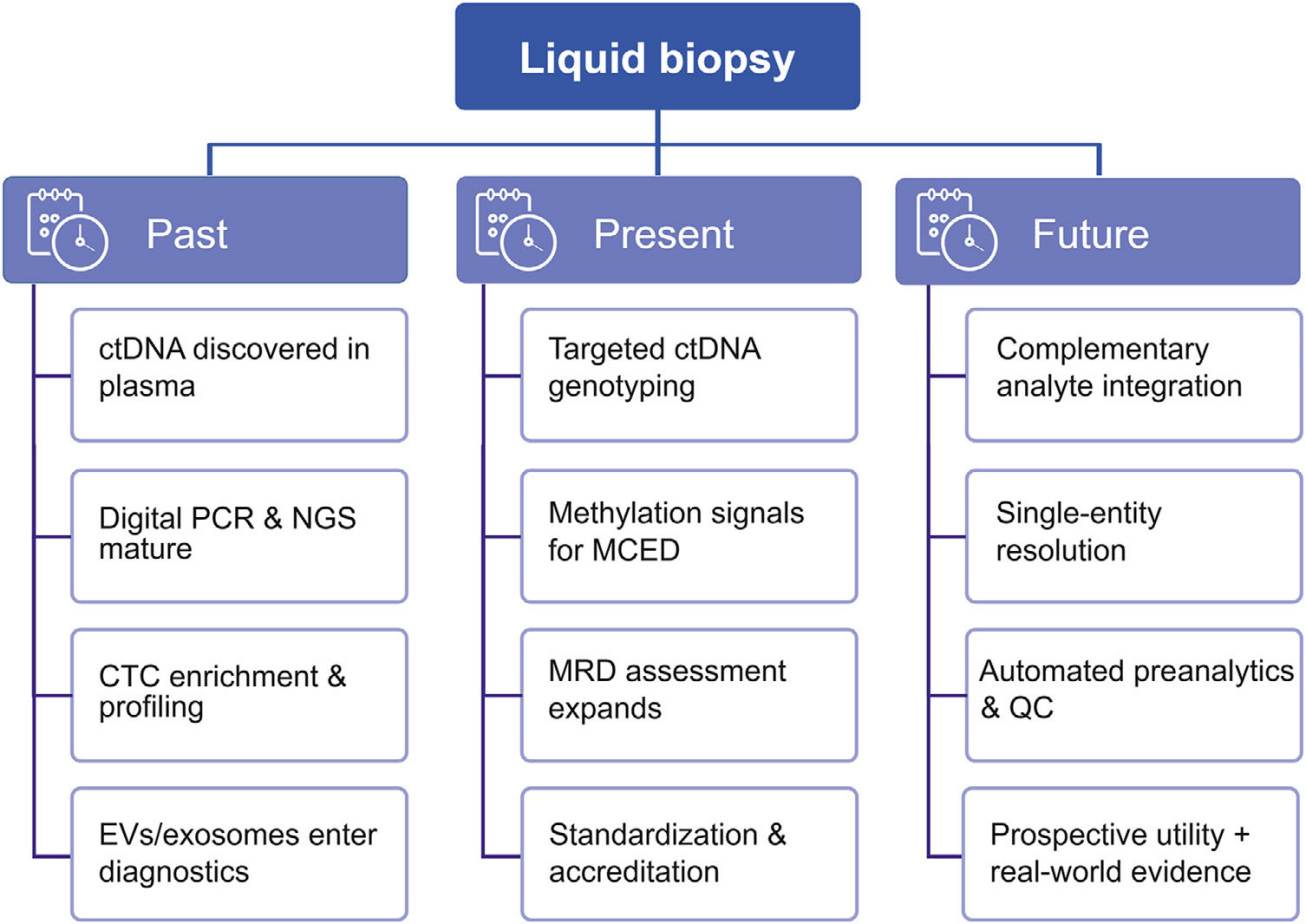
Historical evolution of liquid biopsy from circulating nucleic‐acid discovery to multimodal clinical applications. The timeline highlights the emergence of cfDNA/ctDNA, CTCs, circulating RNAs, extracellular vesicles, and tumor‐educated platelets alongside advances in PCR, sequencing, methylation profiling, fragmentomics, and multiomic analysis. These developments progressively expanded liquid biopsy from analyte detection to early diagnosis, molecular profiling, MRD assessment, and longitudinal monitoring.

### 2.1 CfDNA/ctDNA: From Discovery to Clinically Actionable Molecular Readouts

#### 2.1.1 Evolutionary Overview

cfDNA was first identified in 1948 by Mandel and Métais in human plasma, in both healthy individuals and patients with diverse diseases [14]. This seminal finding established the existence of circulating nucleic acids and laid the foundation for decades of investigation into their biological origins and clinical relevance.

In 1977, Leon et al. reported significantly elevated cfDNA concentrations in the serum of cancer patients compared with healthy controls [15], linking cfDNA abundance to malignant disease states and motivating broader exploration across infectious and inflammatory conditions. These early studies collectively positioned cfDNA as a systemic marker of tissue injury and cell turnover rather than a tumor‐specific signal.

A key conceptual breakthrough occurred in 1994 when KRAS mutations were detected in plasma cfDNA from a pancreatic cancer patient [16]. This provided direct evidence that a fraction of cfDNA originates from tumor cells, formally establishing the concept of ctDNA. The clinical scope of cfDNA expanded further in 1997, when Lo and colleagues discovered fetal DNA sequences in maternal plasma [17], which catalyzed noninvasive prenatal testing (NIPT) and demonstrated that cfDNA can enable highly impactful clinical applications outside oncology [18].

At the methodological level, the introduction of massively parallel sequencing in 2011 accelerated NIPT adoption by improving throughput and reducing cost [19], while simultaneously enabling increasingly sensitive ctDNA analysis in cancer. By 2014, ctDNA assays were clinically validated for KRAS/BRAF detection in colorectal cancer (CRC), marking a transition from proof‐of‐concept to clinical translation [20]. Subsequent years witnessed a shift beyond mutation‐centric assays toward epigenetic and genome‐wide approaches, including methylation‐based MCED platforms such as those developed by Grail/Galleri [21], and multianalyte strategies such as CancerSEEK [22]. In parallel, cfDNA whole‐genome sequencing‐based strategies were evaluated in multicenter early‐detection programs for hepatocellular carcinoma (HCC), including the PreCar project [23]. More recently, Mutation Capsule Plus technology further advanced blood‐based early detection efforts [24].

#### 2.1.2 Essential Connotation and Clinical Relevance

cfDNA refers to extracellular DNA fragments circulating in plasma. In healthy individuals, cfDNA primarily derives from physiological cell turnover (apoptosis, necrosis, pyroptosis) and active release processes (NETosis, EV‐associated release) [25]. Because cfDNA can originate from virtually any tissue (and even microorganisms), it provides a real‐time “snapshot” of systemic cell death dynamics.

cfDNA fragments are typically ∼160 nucleotides in length, consistent with nucleosomal packaging. Their persistence in circulation is short (5–150 min), influenced by protection from histone complexes, lipids, or vesicular association [26, 27]. This rapid turnover underpins cfDNA's utility for dynamic monitoring, but it also imposes stringent preanalytical and analytical requirements.

ctDNA is the tumor‐derived fraction of cfDNA and reflects tumor‐specific genomic and epigenomic alterations. Its abundance depends on tumor burden, location, vascularity, metastatic status, and stage, leading to highly variable fractions (<0.1 to >90%) [17, 20]. Although most ctDNA testing uses plasma, tumor‐derived DNA can also be obtained from ascites, pleural effusion, urine, and cerebrospinal fluid (CSF), with CSF being particularly valuable for CNS malignancies.

The short half‐life of ctDNA (approximately 15 min to 2.5 h) enables real‐time tracking of tumor dynamics—often earlier than conventional protein biomarkers that may require weeks to rise [28]. Beyond somatic mutations, ctDNA carries information on methylation, microsatellite instability (MSI), and other tumor‐linked features. Notably, ctDNA fragments in cancer patients tend to be shorter than background cfDNA (often by ∼20–50 bp), a property that can be exploited to enrich tumor signal [29].

A central quantitative metric is the variant allele fraction (VAF), defined as the fraction of cfDNA molecules harboring a specific mutation at a genomic locus. Accurate detection and quantification of low VAF mutations remains a major technical bottleneck [30]. This challenge stems from cfDNA's short fragment length, low plasma concentration (∼2.5–10 ng/mL), and the overwhelming wild‐type background, which together impede discrimination of rare mutant alleles [31]. Accordingly, high‐sensitivity detection strategies often incorporate enrichment and error suppression.

At the analytical level, current ctDNA technologies typically rely on amplification‐based approaches and/or targeted sequencing. qPCR enables real‐time amplification monitoring and multiplexing [32], while digital PCR provides robust rare‐variant detection with precise absolute quantification [33, 34]. Next‐generation sequencing (NGS) supports broader genomic coverage, including coding and noncoding regions and structural variants [35, 36, 37]. ARMS offers rapid, cost‐effective single‐site mutation screening with reliable detection of low‐frequency actionable mutations, supporting treatment stratification [38, 39].

cfDNA and ctDNA established the molecular foundation of liquid biopsy by enabling minimally invasive, real‐time interrogation of tumor‐derived genomic and epigenomic alterations. However, their acellular, fragmented, and population‐averaged nature—and their strong dependence on tumor shedding—fundamentally constrain sensitivity and biological interpretability, particularly in early‐stage disease. These intrinsic limitations motivated a conceptual shift toward cellular analytes, most notably CTCs, which preserve intact genomes, transcriptomes, and phenotypic states and enable functional interrogation beyond fragmented nucleic acids.

### 2.2 CTCs: Adding a Cellular Dimension to Liquid Biopsy

#### 2.2.1 Evolutionary Overview

The concept of CTCs traces back to 1869, when Ashworth observed tumor cells in the blood of a patient with metastatic cancer [40]. However, progress remained slow for more than a century, largely due to the extreme rarity of CTCs and the technical difficulty of enrichment and detection.

In 1998, Racila et al. reported the isolation of CTCs from blood and demonstrated correlations with pathological staging, marking a modern resurgence and laying groundwork for clinical exploration [41]. Subsequent studies established CTC enumeration as a prognostic biomarker, particularly in metastatic breast cancer (BC), where baseline CTC counts predicted overall and progression‐free survival (PFS). Later work reinforced these associations across cohorts, including links between increased CTC counts and reduced PFS [42].

Importantly, standardization and clinical recognition progressed over time, with AJCC recommendations addressing CTC testing for prognostic assessment in BC [43], and incorporation into national guidelines in China [44]. Despite expanding clinical interest, standardized clinical CTC enumeration remains centered on platforms such as CellSearch, underscoring both the maturity and the ongoing bottlenecks of the field [45, 46].

#### 2.2.2 Essential Connotation and Clinical Relevance

CTCs are tumor cells detached from primary or metastatic sites that enter peripheral blood spontaneously or through iatrogenic processes. These cells typically survive only 1–2.5 h in circulation before immune clearance [47, 48]. Nevertheless, a subset can evade apoptosis/phagocytosis, undergo epithelial–mesenchymal transition (EMT), and contribute to distant metastasis [49, 50].

CTCs provide unique value as cellular analytes: they contain intact DNA, RNA, and proteins and preserve spatial and phenotypic information (e.g., nuclear vs. cytoplasmic localization), which is inaccessible to ctDNA and many EV‐derived signals [51, 52]. As viable entities, CTCs can capture dynamic tumor biology and heterogeneity more directly, supporting applications in early diagnosis, metastasis risk prediction, recurrence monitoring, and treatment response evaluation [53].

At the biological and technical levels, two major barriers constrain broader clinical translation: extreme rarity and high heterogeneity (including EpCAM‐low/negative subpopulations). These challenges have driven development of enrichment and detection technologies.

#### 2.2.3 Enrichment and Detection

CTC enrichment methods are broadly divided into immunoenrichment and physical enrichment, with the shared goal of reducing background blood cells to facilitate downstream analysis [54].

Immunoenrichment includes immunomagnetic bead‐based capture and microfluidic immunoadsorption chips. Positive enrichment commonly targets epithelial markers such as EpCAM [55]. CellSearch is a representative immunomagnetic positive‐enrichment platform for clinical CTC enumeration [46]. Negative enrichment removes leukocytes to enrich CTCs in a marker‐agnostic manner [56]. Microfluidic immunoadsorption offers rapid processing and tunable flow patterns to enhance antibody–cell interactions [57].

Physical enrichment exploits intrinsic differences between CTCs and blood cells (size, density, surface charge) [58]. Filtration‐based devices and size‐selective materials are widely explored, though trade‐offs exist between capture efficiency and cell integrity.

CTC detection aims to confirm presence/abundance and characterize molecular subtypes. Common methods include immunofluorescence labeling, RNA in situ hybridization, qPCR‐based assays, and flow cytometry [59, 60]. Emerging approaches such as imaging‐based analysis, mutation profiling, and single‐cell sequencing further enhance biological resolution, and multiplex immunofluorescence panels have been increasingly used to map CTC subtype distributions [61, 62].

CTCs broaden liquid biopsy from molecular fragments to intact cells, but their rarity and technical demands limit scalability—especially for early detection. This gap has propelled interest in abundant, stable circulating signals such as ncRNAs and EVs, which can amplify tumor‐derived information via protected extracellular compartments and host‐response signatures.

### 2.3 ncRNAs: From “Transcriptional Noise” to Circulating Regulatory Biomarkers

#### 2.3.1 Evolutionary Overview

Studies demonstrating stable circulating miRNAs and their ability to distinguish patients with cancer from controls helped establish ncRNAs as candidate liquid‐biopsy biomarkers [63]. Genome‐wide characterization subsequently established that circular RNAs (circRNAs) are abundant and conserved, broadening interest in this RNA class [64]. Standardization of nomenclature and database resources further accelerated the field [65].

#### 2.3.2 Essential Connotation and Clinical Relevance

ncRNAs constitute a major component of circulating cell‐free RNA (cfRNA), alongside coding RNAs such as mRNA [66]. Circulating ncRNAs can be actively secreted or released upon cell death and play central roles in regulating chromatin state, transcription, and cell fate programs [67]. Their stability in blood, tissue‐associated expression patterns, and potential to discriminate malignant from premalignant states support their value as liquid biopsy biomarkers [68, 69].

miRNAs (typically 18–25 nt) regulate gene expression through RISC complexes by inhibiting translation or promoting target mRNA degradation [70, 71]. A substantial population of circulating miRNAs is associated with Argonaute2 complexes independently of vesicles, which can protect these RNAs from extracellular degradation [72]. Numerous miRNAs exhibit cancer‐associated differential expression, supporting use in diagnosis, monitoring, and prognosis; panels have been proposed across cancers including lung cancer and HCC [73, 74].

Methodologically, miRNA detection includes sequencing‐based (small RNA‐seq), amplification‐based (qRT‐PCR), and hybridization‐based (microarrays, NanoString nCounter, ISH) approaches, each with distinct strengths and limitations in multiplexing, sensitivity, and clinical scalability [75, 76, 77, 78]. Long ncRNAs (lncRNAs; >200 nt) contribute to transcriptional regulation and chromatin remodeling and show circulating biomarker potential in multiple cancers, including HCC and lung cancer [79]. circRNAs and snoRNAs further expand the ncRNA biomarker landscape [80, 81].

Although circulating ncRNAs capture dynamic regulatory states, their extracellular stability, cellular provenance, and susceptibility to preanalytical variability remain incompletely resolved. These limitations have catalyzed growing interest in EVs, which encapsulate nucleic acids and proteins within protected lipid bilayers and provide a more stable and biologically contextualized source of circulating information.

### 2.4 EVs: Protected Cargo and Intercellular Communication

#### 2.4.1 Evolutionary Overview

EVs were first observed in 1983 and later termed “exosomes/EVs” in the late 1980s but were initially viewed as cellular waste [82, 83]. A major turning point came in 2007 when Valadi et al. demonstrated that EVs carry functional mRNA and miRNA, revealing a mechanism for intercellular genetic communication and revitalizing the field [84]. Subsequent recognition of vesicle trafficking mechanisms further brought EV research to the mainstream, while standardization efforts (ISEV nomenclature and guidance) improved methodological rigor.

#### 2.4.2 Essential Connotation and Clinical Relevance

EVs are nanoscale phospholipid bilayer vesicles released by living cells and enriched in diverse cargo (mRNA, miRNA, lncRNA, DNA species, proteins). They are present in many biofluids (blood, saliva, BAL fluid, sputum) [85]. Tumor cells can release large quantities of EVs, potentially linked to MAPK pathway activity; these vesicles participate in tumorigenesis and progression and provide protected compartments for biomarker detection [86, 87].

EV cargo can enhance nucleic acid stability and enable complementary profiling of tumor biology. For example, EV‐associated miRNAs have shown diagnostic potential in lung cancer and lung adenocarcinoma (LUAD) discrimination [88]. However, clinical translation is constrained by variability in EV abundance, separation hardware, purification quality, and the difficulty of isolating low‐abundance vesicles from complex biofluids [85].

Current EV isolation methods include density‐based separation, polymer precipitation, size‐based approaches (ultrafiltration), affinity capture (immunoaffinity chromatography), electrodynamic separation, size‐exclusion chromatography, ultracentrifugation, and microfluidics [89]. Each method entails trade‐offs (e.g., ultracentrifugation is time‐consuming; ultrafiltration risks contamination/denaturation; immunoaffinity capture can yield low amounts). Microfluidics offers portability, speed, and lower contamination risk, supporting translational potential [90]. Given limitations of single methods, integrated workflows are often required.

EV characterization commonly relies on vesicle tracing, transmission electron microscopy, and particle sizing [91]. Downstream cargo analysis typically employs immunoblotting, ELISA, and flow cytometry for protein profiling, and qRT‐PCR, dPCR, NGS, microarrays, Northern blotting, or FISH for RNA analysis. Emerging biosensor platforms, including electrochemical, colorimetric, and nano‐biosensor‐based assays, further enhance analytical sensitivity.

EVs offer protected, information‐rich cargo that bridges molecular and cellular liquid biopsy paradigms by coupling nucleic acid signals with their cellular context. However, pronounced heterogeneity in vesicle populations, the absence of standardized isolation and characterization protocols, and the scarcity of clinically validated, scalable assays currently impede large‐scale implementation, positioning EVs as powerful yet methodologically demanding analytes.

Beyond serving as tumor‐derived analytes, EVs also mediate bidirectional communication between tumors and host cells, thereby reshaping systemic circulating components. Among these host‐response signals, TEPs represent an accessible and biologically responsive surrogate of tumor–host interactions.

### 2.5 TEPs: Host‐Response Signals with High Accessibility

TEPs, also termed tumor‐conditioned platelets, refer to platelets whose RNA and protein compositions are dynamically altered by tumor‐derived molecules [92, 93]. Through direct and indirect interactions, cancer cells can induce tumor cell‐induced platelet aggregation, promoting angiogenesis and metastasis and associating with adverse clinical outcomes [94]. Despite being anucleate and short‐lived, TEPs actively participate in multiple cancer hallmarks, including immune evasion and tumor microenvironment remodeling [95].

TEPs have demonstrated diagnostic and prognostic potential across a broad range of solid tumors. Landmark RNA‐sequencing studies achieved high accuracy in distinguishing cancer patients from healthy individuals and provided tumor‐type localization signals, including discrimination of oncogenic drivers such as KRAS and EGFR [93]. In non‐small cell lung cancer (NSCLC), TEP‐based assays exhibited stage‐dependent performance, and platelet ITGA2B expression has been associated with prognosis and pulmonary nodule stratification [96]. Additional evidence supports TEP alterations in HCC, glioma, BC, and CRC, including TIMP1 mRNA as a candidate biomarker in CRC [97]. Compared with CTCs and ctDNA, TEPs are relatively easy to access and isolate, supporting scalable assay development.

TEP analyses commonly use RNA sequencing to identify platelet transcriptomic signatures, with targeted RT‐PCR used to validate selected RNA markers [93, 96, 98]. However, several challenges currently limit clinical translation, including incomplete mechanistic understanding of tumor–platelet interactions, limited integration of platelet transcriptomic and proteomic data (given partial translation rates), and the need for larger, tumor‐type‐specific validation cohorts [99].

TEPs capture systemic tumor–host crosstalk and provide highly accessible RNA‐based biomarkers with promising diagnostic and tumor‐localization capabilities. Nevertheless, unresolved biological mechanisms and insufficient multiomics integration continue to impede clinical standardization, underscoring the necessity of rigorous preanalytical control as liquid biopsy platforms expand across diverse analytes.

### 2.6 Preanalytical Standardization: From Analytical Potential to Clinical Reliability

Preanalytical standardization is essential for translating liquid biopsy from research to routine clinical practice, as inconsistent sample handling can generate artifactual signals and undermine reproducibility [100]. Because liquid biopsy analytes (cfDNA/ctDNA, CTCs, EVs) differ in stability and sensitivity to processing, harmonized procedures for collection, stabilization, centrifugation, storage, and documentation are indispensable [101, 102].

Peripheral blood is the most common cfDNA source, though saliva, ascites, and urine may be informative in specific settings. Plasma is generally preferred over serum due to lower risk of genomic DNA contamination from leukocyte lysis [103]. For CTCs, circadian variation in CTC release has motivated standardized sampling recommendations (e.g., morning fasting sampling with time annotation) to reduce biological variability [104, 105].

Tube selection is critical given cfDNA's short half‐life. EDTA tubes require timely processing to prevent leukocyte lysis and gDNA contamination; stabilized cfDNA tubes (e.g., Streck) extend allowable processing windows and preserve ctDNA integrity under ambient conditions. Centrifugation workflows (two‐step spins) and low‐temperature control reduce cellular carryover and degradation, and long‐term storage is typically at –80°C [106, 107, 108, 109].

Regulatory and quality frameworks (CLIA/CAP, ISO 15189), external quality assessment schemes, and global consortia (BloodPAC, CANCER‐ID, ELBS, ILSA) have played major roles in developing reference materials, benchmarking performance, and establishing best‐practice guidelines. Remaining challenges include consensus on processing delays for different analytes and further standardization for emerging biomarkers such as EV‐derived DNA/RNA [13, 110, 111, 112].

At the analytical level, preanalytical standardization is the critical enabler that transforms liquid biopsy from a high‐potential research tool into a clinically reliable diagnostic modality. Harmonized protocols for sample collection, processing, and quality control are indispensable for reproducibility, regulatory approval, and cross‐platform comparability [113, 114].

Collectively, the historical evolution of liquid biopsy reflects a progressive layering of biological information—from fragmented nucleic acids to intact cells, vesicular cargo, and host‐response signals. Each analyte class addresses distinct biological and technical limitations, underscoring that clinical utility arises not from a single biomarker, but from context‐dependent integration of complementary liquid biopsy modalities. These advances now underpin a rapidly expanding spectrum of clinical applications across oncology and nononcologic diseases.

## 3 Oncologic Applications: Clinical Use Cases and Evidence Maturity

Liquid biopsy has transitioned from an analytical innovation to a clinically actionable modality in cancer care. However, its clinical utility is inherently context dependent, shaped by tumor biology, anatomical constraints, stage‐specific tumor burden, and the specific clinical questions being addressed. Different liquid biopsy analytes—including ctDNA, CTCs, EVs, ncRNAs, and host‐response signals—exhibit distinct strengths and limitations that manifest differently across cancer types.

In cancer care, liquid biopsy has demonstrated particular value in early detection, molecular profiling for targeted therapy selection, MRD assessment, treatment response monitoring, and resistance tracking. Importantly, no single analyte is universally optimal. Instead, effective clinical deployment relies on aligning specific analytes with appropriate clinical use cases and tumor contexts. In this section, we provide a system‐based overview of liquid biopsy applications across major cancer contexts, highlighting how different analytes address distinct unmet clinical needs.

To distinguish clinically established applications from emerging approaches, representative liquid‐biopsy use cases are classified into four evidence‐maturity tiers throughout this section: Tier 1, clinically established, comprising applications supported by regulatory approval and/or professional guideline recognition in defined clinical settings; Tier 2, late‐stage clinical translation, supported by substantial prospective, multicenter, or otherwise advanced clinical‐validation evidence but not yet routinely implemented; Tier 3, early‐stage clinical validation, encompassing applications evaluated in clinical cohorts but still requiring broader prospective or external validation; and Tier 4, discovery and mechanistic exploration, including early exploratory human studies and predominantly mechanistic or preclinical evidence. Importantly, tiers are assigned to specific clinical use case–assay combinations rather than to analytes themselves because the same analyte may have markedly different levels of maturity across indications.

### 3.1 Lung Cancer

#### 3.1.1 Disease Background and Unmet Clinical Needs

Lung cancer remains among the most common malignancies globally and has a high mortality rate [115]. It is broadly divided into small cell lung cancer and NSCLC, with NSCLC accounting for about 85% of cases and including LUAD and squamous cell carcinoma (LUSC) [32, 116]. Intratumoral and intertumoral heterogeneity make NSCLC difficult to diagnose and treat.

Approximately 60% of patients present with distant metastases at the time of clinical diagnosis, resulting in poor overall survival (OS) [117]. Although early detection and intervention substantially improve outcomes, lung cancer is characterized by a prolonged asymptomatic phase, often spanning several years, which limits the effectiveness of symptom‐driven diagnosis.

Conventional diagnostic modalities suffer from inherent limitations, including the invasiveness and procedural risks of percutaneous transthoracic needle aspiration, the limited sensitivity and high false‐positive rates of low‐dose computed tomography for subcentimeter nodules, and the inability of single‐site tissue biopsies to capture intratumoral heterogeneity—an increasingly critical barrier in precision oncology [118]. Liquid biopsy now supports molecular profiling and longitudinal monitoring in advanced NSCLC and is being actively evaluated for early detection and screening.

#### 3.1.2 Early Detection and Screening

ctDNA has been extensively evaluated for early lung cancer detection [119]. Plasma ctDNA levels are significantly elevated in lung cancer patients compared with individuals with benign inflammatory lung diseases, suggesting potential value in distinguishing malignant from nonmalignant respiratory conditions; prospective validation in screening populations is needed.

Plasma DNA‐based approaches have demonstrated diagnostic potential in lung cancer, although sensitivity remains strongly dependent on disease stage, tumor shedding, and assay design [120, 121].

The diagnostic and prognostic value of ctDNA is grounded in the detection of tumor‐specific molecular alterations, including somatic mutations, loss of heterozygosity (LOH), MSI, and aberrant DNA methylation patterns [120, 122]. These features make ctDNA a minimally invasive molecular surrogate for early diagnosis and risk stratification.

However, in early‐stage NSCLC, interpretation of ctDNA mutations requires caution, as low‐frequency variants may originate from non‐neoplastic clonal hematopoiesis or reflect early tumorigenic processes with uncertain clinical significance [123]. Accordingly, comprehensive analytical frameworks and conservative clinical interpretation are essential when leveraging ctDNA for early detection.

#### 3.1.3 Molecular Profiling and Therapy Selection

Beyond early detection, ctDNA analysis is central to managing advanced and metastatic lung cancer. In this setting, liquid biopsy supports molecular profiling for therapy selection, prognosis, and dynamic monitoring of response and resistance [124].

Among the most well‐established applications is the detection of EGFR mutations in ctDNA. Blood‐based ctDNA assays reliably identify EGFR mutational status and have demonstrated strong concordance with tumor tissue genotyping, while also capturing spatial and temporal tumor heterogeneity. Clinically, ctDNA‐detected EGFR mutations correlate with disease progression and patient outcomes; notably, the L858R subtype has been associated with significantly shorter OS compared with other EGFR genotypes, underscoring its prognostic relevance [125]

#### 3.1.4 MRD Assessment and Recurrence Prediction

Serial ctDNA profiling has emerged as a powerful tool for postoperative MRD surveillance and recurrence prediction in early‐stage NSCLC. Detection of ctDNA following curative‐intent surgery identifies patients at markedly elevated risk of radiographic recurrence, with lead times ranging from several months to over a year [122].

Landmark studies have provided strong evidence supporting the prognostic value of ctDNA MRD in NSCLC. In the TRACERx study, landmark MRD analysis achieved a positive predictive value (PPV) of 93% for recurrence, with a median lead time of 228 days [126]. In the NADIM II trial, MRD positivity was associated with a hazard ratio of 10.2 for event‐free survival and OS, while consecutive MRD‐negative samples identified non‐pCR patients with favorable outcomes [127]. These findings highlight ctDNA MRD as a prognostic biomarker that warrants evaluation in prospective interventional trials.

#### 3.1.5 Treatment Response and Resistance Monitoring

Serial ctDNA profiling further enables real‐time assessment of treatment response and early identification of acquired drug resistance. Emerging resistance‐associated mutations—including previously unrecognized subclonal variants arising under therapeutic pressure—can be detected prior to radiographic progression, providing an earlier molecular signal of emerging resistance, particularly in targeted‐therapy settings [122].

Clinical implementation of plasma ctDNA testing in advanced NSCLC is reflected in guideline‐supported molecular testing. Plasma genotyping is incorporated into advanced NSCLC workflows for selected actionable alterations in appropriate clinical settings [128, 129].

#### 3.1.6 Prognostic Stratification

Beyond therapy selection, ctDNA and CTCs provide complementary prognostic information in lung cancer. In advanced NSCLC, ctDNA‐detected EGFR mutations, particularly the L858R subtype, have been associated with shorter OS compared with other EGFR genotypes, underscoring their prognostic relevance [125].

CTCs are detectable across all stages of lung cancer progression and represent a key cellular substrate underlying distant metastasis. However, owing to their extreme rarity in peripheral blood during early tumor development, reliable CTC detection is considerably more feasible in advanced‐stage disease, where hematogenous dissemination is more pronounced.

In early‐stage lung cancer, low CTC abundance has historically limited their diagnostic sensitivity. Recent technological advances have partially overcome these challenges. Notably, the integration of CellCollector in vivo CTC capture technology with downstream CTC‐based NGS has achieved a detection rate of 62.5% in patients with Stage I–II NSCLC, representing a substantial improvement over conventional ex vivo enrichment approaches [130].

Beyond enumeration, molecular profiling of CTCs has expanded their diagnostic and biological relevance. NGS analysis of CTCs has identified several high‐frequency mutated genes—including NOTCH1, IGF2, EGFR, and PTCH1—that may serve as candidate markers for early lung cancer detection [131, 132]. In addition, metabolomic profiling of CTCs has revealed dozens of differentially expressed metabolites, with a subset demonstrating potential clinical value for distinguishing early‐stage lung cancer from nonmalignant conditions [133].

Importantly, CTCs are exceedingly rare in the blood of nontumor‐bearing individuals, conferring high analytical specificity. This intrinsic specificity underscores their value as complementary biomarkers for early detection and metastatic risk assessment. Nevertheless, further optimization of enrichment efficiency, phenotypic coverage, and analytical standardization remains necessary to enhance sensitivity and enable broader clinical implementation.

#### 3.1.7 Emerging Biomarkers and Multianalyte Integration

##### 3.1.7.1 Noncoding RNAs

Circulating ncRNAs—including microRNAs (miRNAs), lncRNAs, and circRNAs—have become useful markers in lung cancer liquid biopsy, reflecting tumor progression, metastatic potential, and therapeutic response [134].

Among miRNAs, let‐7a is one of the most extensively studied biomarkers, with significantly reduced serum levels observed in NSCLC patients compared with healthy individuals [135]. miR‐125 has also demonstrated diagnostic value, achieving a sensitivity of 82% and specificity of 77% by quantitative PCR‐based detection. Beyond individual markers, multi‐miRNA signatures provide enhanced prognostic resolution. For example, an eight‐miRNA model (miR‐1260b, miR‐21‐3p, miR‐92a‐3p, miR‐2467‐3p, miR‐4659a‐3p, miR‐4514, miR‐1471, and miR‐3621) has been developed to assess lung cancer prognosis and metastatic clinical features [136, 137].

LncRNAs and circRNAs further extend the regulatory landscape of lung cancer liquid biopsy. Prognostic models incorporating multiple lncRNAs have demonstrated utility in evaluating metastatic risk and clinical outcomes. In parallel, several circRNAs—including hsa_circ_0062682, hsa_circ_0092283, and hsa_circ_0070610—have been identified as potential candidates for prognostic stratification in lung cancer [138].

##### 3.1.7.2 Extracellular Vesicles

EVs, particularly exosomes, provide an additional layer of biological information by protecting and transporting tumor‐derived RNA and protein cargo. Lung cancer‐derived exosomes contribute to immunosuppressive microenvironment formation and tumor proliferation. Notably, exosomal PD‐L1 inhibits CD8^+^ T cell activity, facilitating immune evasion. Exosome‐associated PD‐L1 levels have been associated with treatment response and prognosis in clinical cohorts of NSCLC patients receiving immune checkpoint inhibitors [139, 140].

Multianalyte exosomal RNA signatures further enhance diagnostic performance. Distinct exosomal miRNA panels can discriminate LUAD from benign pulmonary granulomas, differentiate lung cancer patients from healthy individuals, and stratify NSCLC subtypes. For example, plasma exosomes enriched in miR‐30e‐3p and miR‐181‐5p are specific for adenocarcinoma, whereas miR‐15b‐5p and miR‐10b‐5p are associated with squamous cell carcinoma [141].

Beyond their diagnostic role, EVs actively participate in therapy resistance. Cisplatin‐treated A549 lung cancer cells exhibit increased exosome secretion and altered exosomal miRNA and mRNA profiles related to drug sensitivity [142, 143]. These exosomes can confer cisplatin resistance to recipient cells, suggesting that inhibition of exosome biogenesis or release may enhance chemosensitivity and represent a novel therapeutic strategy.

##### 3.1.7.3 Tumor‐Educated Platelets

TEPs have recently gained attention as accessible and biologically responsive liquid biopsy analytes in lung cancer. Through direct and indirect interactions with tumor cells, platelets acquire tumor‐derived RNA signatures that reflect disease presence and progression.

RNA sequencing of platelet samples from patients with early‐stage NSCLC has revealed significantly elevated expression of ITGA2B compared with healthy controls. TEP‐derived ITGA2B has shown diagnostic and prognostic associations in clinical cohorts, including potential utility for Stage I NSCLC and pulmonary‐nodule stratification, although broader external and prospective validation is required before clinical implementation. Moreover, when combined with carcinoembryonic antigen (CEA) levels and tumor stage, ITGA2B serves as an independent predictor of OS in NSCLC patients [96].

#### 3.1.8 Evidence Maturity and Translational Status

Liquid biopsy applications in lung cancer span four levels of evidence maturity (Table 1). Tier 1—clinically established is concentrated in plasma ctDNA‐based targeted testing and comprehensive genomic profiling for actionable alterations in defined advanced or metastatic NSCLC settings. Guideline‐supported plasma molecular testing is incorporated into these workflows, although low tumor shedding means that negative plasma results may still require tissue confirmation. Tier 2—late‐stage clinical translation includes postoperative ctDNA MRD assessment and cfDNA fragmentomics for early detection or pulmonary‐nodule risk stratification. Both have prospective or multicohort validation, whereas routine MRD‐directed treatment decisions and population‐level screening remain unestablished. Tier 3—early‐stage clinical validation includes cfDNA methylation classifiers, TEP RNA signatures, CTC/CTEC assays, and exosomal miRNA profiles supported by clinical‐cohort evidence but with incomplete external validation and standardization. Tier 4—discovery and mechanistic exploration comprises EV‐associated ncRNA and circRNA pathways investigated predominantly in mechanistic models of treatment resistance and immune escape.

**TABLE 1:** Evidence maturity of representative liquid biopsy applications in lung cancer.

Evidence tier	Clinical application	Representative analyte/assay strategy	Sample source	Disease context	Key representative evidence	Validation status	Regulatory/guideline status	Major limitations
Tier 1—clinically established	Molecular profiling and therapy selection; resistance monitoring	Plasma ctDNA targeted testing/comprehensive genomic profiling for actionable drivers (e.g., EGFR, KRAS G12C, ERBB2, BRAF, MET, RET, ALK/ROS1/NTRK‐related alterations)	Plasma	Advanced/metastatic NSCLC; initial molecular work‐up and selected progression settings	Plasma genotyping is integrated into advanced NSCLC workflows for selected actionable alterations [128, 129].	Prospective validation and guideline‐supported clinical implementation in defined indications	Guideline‐supported in defined advanced NSCLC settings; use remains biomarker and context specific.	A negative plasma result may require tissue testing because of low shedding; histologic transformation is not reliably captured; preanalytical and clonal‐hematopoiesis confounding remain relevant.
Tier 2—late‐stage clinical translation	MRD assessment and recurrence prediction	Tumor‐informed and tumor‐naive ctDNA MRD assays	Plasma; tumor tissue may be required for tumor‐informed assays	Resectable early‐stage NSCLC after surgery and/or perioperative therapy	TRACERx linked ctDNA MRD to metastatic relapse and provided molecular lead time; perioperative studies show strong prognostic stratification beyond pathologic response [126, 127].	Prospective multicenter observational cohorts and prospective trial cohorts	Investigational for MRD‐directed treatment decisions; commercial assays may be available, but routine escalation/de‐escalation based solely on MRD is not established.	Low‐shedding tumors can remain ctDNA‐negative; optimal sampling and action thresholds are unresolved; prognostic validity does not by itself establish treatment utility.
Tier 2—late‐stage clinical translation	Early detection/pulmonary nodule risk assessment	cfDNA fragmentomics and related genome‐wide features	Plasma cfDNA	Early‐stage lung cancer and high‐risk/pulmonary‐nodule populations	Lung cancer‐specific fragmentomic studies demonstrated discrimination and histologic information in independent cohorts [121].	Multicohort clinical validation; not population‐scale screening utility evidence	Investigational; no routine guideline‐supported population screening role	Performance depends on stage and tumor shedding; false positives and downstream work‐up matter; prospective screening utility and cost effectiveness remain unproven.
Tier 3—early‐stage clinical validation	Early detection/nodule stratification	cfDNA methylation panels (e.g., 7‐DMR and LUNG–TRAC‐type classifiers)	Plasma cfDNA	Pulmonary nodules and early‐stage NSCLC	Prospective and independent‐cohort studies reported useful discrimination, but performance varied across cohorts [144, 145].	Prospective single‐center to multicenter clinical validation	Investigational/research‐use; no routine screening recommendation	Small‐to‐moderate cohorts, population restriction, assay‐specific cutoffs, and reduced sensitivity in external or multicenter testing.
Tier 3—early‐stage clinical validation	Early diagnosis and prognostic stratification	Tumor‐educated platelet RNA (ITGA2B) and CTC/CTEC‐based assays	Platelets/peripheral blood	Early and advanced NSCLC	Platelet ITGA2B and CTC/CTEC studies support diagnostic or prognostic associations in clinical cohorts [96, 146].	Retrospective and early prospective clinical validation	Investigational	Low abundance, platform heterogeneity, uncertain tumor specificity, and limited external validation.
Tier 3—early‐stage clinical validation	Early diagnosis/histologic subtyping	Tumor‐derived exosomal miRNA signatures	Plasma/serum EVs	Early‐stage NSCLC and histologic subtypes	Exosomal miRNA profiling has shown diagnostic signals in patient cohorts [147].	Retrospective clinical validation with limited external validation	Investigational	EV isolation and normalization are not standardized; small cohorts and case–control designs may inflate performance.
Tier 4—discovery and mechanistic exploration	Mechanistic resistance and immune‐evasion biology	Exosomal ncRNAs and circRNAs (e.g., miR‐4443, circUSP7 and related pathways)	EVs; cell lines/experimental models with selected patient samples	Therapy resistance and immune escape in NSCLC	Mechanistic studies link EV‐associated ncRNAs to cisplatin resistance and anti‐PD‐1 resistance [148, 149].	Predominantly mechanistic/preclinical evidence	Not clinically validated	Mechanistic association does not establish biomarker utility; assay reproducibility, prospective validation, and clinical decision thresholds are lacking.

Abbreviations: CDx, companion diagnostic; cfDNA, cell‐free DNA; CHIP, clonal hematopoiesis of indeterminate potential. Entries are representative rather than exhaustive and are intended to mirror the principal use cases discussed in the corresponding manuscript section; CTC, circulating tumor cell; ctDNA, circulating tumor DNA; CTEC, circulating tumor endothelial cell; EV, extracellular vesicle; MRD, minimal residual disease; NSCLC, non‐small cell lung cancer; TEP, tumor‐educated platelet; VAF, variant allele fraction.

### 3.2 Hepatocellular Carcinoma

#### 3.2.1 Disease Background and Unmet Clinical Needs

HCC is the most common primary liver cancer and a leading cause of cancer‐related death worldwide. It is projected to cause more than 1.3 million deaths annually by 2040 [150]. HCC usually develops in the setting of chronic liver injury and cirrhosis, driven mainly by chronic hepatitis B virus (HBV) or hepatitis C virus (HCV) infection, alcohol‐related liver disease, and the increasingly common metabolic dysfunction‐associated steatotic liver disease [151, 152].

Despite substantial advances in HCC management, major clinical challenges persist. Curative interventions such as surgical resection or ablation are largely restricted to early‐stage disease, whereas advanced HCC relies on systemic therapies, including immunotherapy‐based combinations that have recently become standard of care [153]. However, treatment response and the emergence of resistance remain difficult to predict using conventional molecular biomarkers alone, contributing to heterogeneous clinical outcomes [154].

Against this backdrop, two unmet clinical needs dominate HCC management: (i) improving early detection in high‐risk populations with chronic liver disease, and (ii) refining stage‐specific treatment strategies through improved prediction of therapeutic response and resistance, particularly in advanced disease [155, 156]. Traditional diagnostic tools—including ultrasound and serum alpha‐fetoprotein (AFP)—suffer from limited sensitivity and specificity, especially for small or AFP‐negative tumors [157]. Similarly, reliance on single‐gene mutation testing provides insufficient resolution to guide dynamic treatment adaptation [153]. These limitations have sparked interest in liquid biopsy as a diagnostic and monitoring tool for HCC.

#### 3.2.2 Early Detection and Screening

ctDNA, released from apoptotic or necrotic HCC cells, has emerged as a biomarker for early HCC detection, which strongly affects patient prognosis. Plasma ctDNA levels and tumor‐associated molecular alterations are higher in HCC patients than in individuals with benign chronic liver conditions, including chronic HBV infection, nonalcoholic fatty liver disease, or compensated cirrhosis [158, 159]. This distinction addresses a long‐standing clinical challenge: differentiating malignant liver lesions from benign hepatic disorders.

A systematic review of 15 studies and 3686 patients reported that ctDNA‐based HCC detection had a pooled sensitivity of 0.83, specificity of 0.90, and area under the curve (AUC) of 0.93 [160]. Notably, ctDNA targeting key driver alterations—such as TERT promoter and TP53 mutations—is detectable in more than 75% of plasma samples from patients with Stage I HCC [161, 162]. Seminal proof‐of‐concept studies have shown that integrating trunk mutations, HBV integration breakpoints, clinical variables, and protein markers could enable early‐stage HCC diagnosis in carefully selected high‐risk cohorts, with sensitivities approaching 100% and specificities exceeding 90% [163].

The diagnostic performance of ctDNA in HCC reflects its enrichment for tumor‐specific genetic and epigenetic alterations, including recurrent driver mutations (TERT, CTNNB1, TP53, AXIN1), LOH, MSI, and aberrant DNA methylation patterns involving genes such as ECE1, HOXA1, CLE11A, and EMX1 [164, 165, 166].

Several methylation‐based assays have demonstrated robust early detection performance. The HepaAiQ assay, a 20‐DMR methylation panel, achieved a training AUC of 0.944 (86.0% sensitivity, 92.1% specificity) and a validation AUC of 0.940 (84.4% sensitivity, 90.3% specificity), outperforming AFP and DCP [167]. The epiLiver methylation panel, combined with M4 markers, achieved 79.4% sensitivity for Stage A HCC at 100% specificity [168]. Multimarker methylation panels such as Oncoguard Liver, incorporating HOXA1, EMX1, ECE1, and CLEC11A, demonstrated a cross‐validated AUC of 0.94, significantly outperforming AFP (0.96 vs. 0.80, *p* < 0.0001) [169].

Nevertheless, interpretation of ctDNA signals in early‐stage HCC requires caution. Cirrhosis‐associated hepatocyte injury can contribute ctDNA‐like fragments, and the correlation between ctDNA abundance and very small lesions (≤2 cm) remains weak [170]. Accordingly, multiparametric analytical frameworks and conservative clinical interpretation are essential to mitigate false‐positive detection and overdiagnosis.

#### 3.2.3 Molecular Profiling and Therapy Selection

In advanced or metastatic HCC, targeted ctDNA profiling has demonstrated feasibility for molecular characterization, prognostic stratification, and assessment of treatment response or resistance. Most evidence remains observational, and the limited actionability of recurrent HCC alterations currently restricts routine treatment‐guiding use.

Alterations detected in ctDNA—such as TP53 or MET mutations—have been consistently associated with inferior outcomes, with ctDNA‐positive TP53 mutations predicting significantly shorter OS even in patients receiving chemo‐immunotherapy [171]. Mutational profiling of ctDNA has also identified recurrent driver alterations including CTNNB1 (12–17%), ARID1A/2, AXIN1, and PTEN, which may inform therapeutic stratification [166, 172, 173, 174, 175].

#### 3.2.4 MRD Assessment and Recurrence Prediction

Postoperative ctDNA‐based MRD assessment has shown prognostic value in early prospective studies of HCC, although evidence remains insufficient to support routine MRD‐guided treatment decisions. The SeekInCure assay, a tumor‐naive ctDNA multiomics approach integrating copy‐number alterations, fragment size, and AFP, demonstrated 87.5% preoperative detection in a prospective cohort (*n* = 32), with MRD positivity associated with a hazard ratio of 5.67 for OS (*p* = 0.008). Patients with persistent ctDNA postsurgery experienced 55.6% mortality, whereas those with pre‐ and postoperative ctDNA negativity showed 100% survival [176].

TERT promoter mutations detected in ctDNA have been associated with poorer prognosis and shorter OS in HCC [177, 178].

CTC detection and phenotyping have been associated with recurrence risk and survival outcomes in HCC [179].

Recent technological advances have substantially improved CTC detection sensitivity in HCC. The CanPatrol platform achieved detection rates exceeding 90% in early‐stage HCC cohorts, while RNA in situ hybridization‐based approaches captured CTCs in more than 90% of patients in independent studies [180]. Multimarker panels targeting stem‐like CTCs demonstrated robust diagnostic performance, including in AFP‐negative HCC, with AUC values approaching 0.88 [181].

Beyond enumeration, molecular characterization of CTC subpopulations may provide additional prognostic information. Higher levels of PD‐L1‐positive CTCs have been associated with poorer clinical outcomes in patients with HCC receiving PD‐(L)1 blockade plus targeted therapy [182]. Studies comparing HCC with nonmalignant control groups have also evaluated the diagnostic performance of CTC phenotyping [180, 181].

#### 3.2.5 Treatment Response and Resistance Monitoring

Liquid biopsy enables dynamic detection of resistance‐associated molecular changes during systemic therapy. Emergence of alterations in the EGFR or VEGF signaling pathways, as well as rising TERT mutation levels, can signal impending treatment failure [183]. These findings suggest that serial ctDNA profiling may help characterize emerging resistance and identify molecular subclones under treatment pressure. Prospective interventional evidence for ctDNA‐guided treatment switching is still lacking.

In advanced HCC, ctDNA‐detected alterations—such as TP53 or MET mutations—have been consistently associated with inferior outcomes. PI3K/MTOR pathway mutations have been linked to shorter PFS on TKI therapy, suggesting that ctDNA profiling may inform treatment stratification in research settings [171].

#### 3.2.6 Prognostic Stratification

ctDNA‐based markers provide prognostic information in HCC. TERT promoter mutations detected in ctDNA have been associated with poorer prognosis and shorter OS in HCC [177, 178]. TP53 mutations in ctDNA predict aggressive disease and poor survival [184]. LINE‐1 and Sat2 hypomethylation in plasma cfDNA have been associated with unfavorable outcomes and genomic instability [185, 186].

CTC enumeration and subtyping further refine prognostic stratification. A meta‐analysis of 20 studies involving 1191 patients reported that CTC positivity is linked to poor OS (HR 2.31–3.59) and recurrence‐free survival (RFS) (HR 2.42–3.03) [179]. Higher levels of PD‐L1‐positive CTCs have been associated with shorter PFS and OS in patients receiving PD‐(L)1 blockade plus targeted therapy [182].

#### 3.2.7 Emerging Biomarkers and Multianalyte Integration

ncRNAs—including miRNAs, lncRNAs, and circRNAs—have been widely studied as HCC biomarkers. Several miRNA panels can distinguish HCC from benign liver disease and stratify HBV‐ or HCV‐associated disease states. For example, a 13‐miRNA panel distinguishes HBV infection, HCV infection, and HBV‐positive HCC, while miR‐375 alone achieves AUC values up to 0.96 for HCC detection [187].

LncRNAs such as HULC exhibit strong diagnostic potential, with ultrasensitive electrochemical sensors enabling discrimination between HCC, HBV‐positive patients, and healthy controls [188, 189]. EV‐derived lncRNAs further enhance diagnostic performance: panels including EV‐LINC00853, EV‐DLEU2, EV‐SNHG1, EV‐MALAT1, and EV‐HOTTIP outperform AFP, particularly in early‐stage and AFP‐negative HCC [79].

EVs derived from HCC not only serve as diagnostic and prognostic biomarkers but also represent potential therapeutic targets. EV‐derived RNA and protein signatures have been evaluated for early detection, prognostic stratification, and treatment‐response assessment [190, 191]. The HCC EV ECG score, combining EpCAM^+^CD63^+^, CD147^+^CD63^+^, and GPC3^+^CD63^+^ EV markers, achieved a validation AUC of 0.93, outperforming AFP [192]. EV‐based HCC assays remain at an early clinical‐validation stage, with major sources of variability arising from EV isolation, normalization, and cohort design.

Mechanistic studies of circMTO1 and circSETD3 implicate these RNAs in HCC growth and signaling; current evidence is predominantly tissue and model based [193, 194]. EV‐mediated transfer of resistance‐associated molecules suggests that inhibiting EV biogenesis or uptake may enhance therapeutic sensitivity.

#### 3.2.8 Evidence Maturity and Translational Status

Liquid biopsy applications in HCC span several levels of evidence maturity (Table 2), with no representative Tier 1—clinically established application identified under the framework used in this review. Tier 2—late‐stage clinical translation is represented by plasma ctDNA methylation approaches such as HepaAiQ‐type HCC‐specific panels, supported by multicenter clinical validation. Tier 3—early‐stage clinical validation includes blood‐based methylation classifiers, postoperative tumor‐naive ctDNA MRD assessment, targeted ctDNA mutation profiling, CTC enumeration and phenotyping, and EV‐ or circulating RNA‐based assays. Evidence ranges from prospective cohorts to retrospective studies and meta‐analyses, with assay maturity varying substantially across applications. Tier 4—discovery and mechanistic exploration comprises predominantly circRNA and lncRNA studies based on tumor tissue, cell models, and experimental systems. Overall, methylation‐based approaches currently have the strongest translational evidence in HCC, whereas MRD, mutation profiling, CTC‐, EV‐, and RNA‐based applications remain at earlier stages of clinical development.

**TABLE 2:** Evidence maturity of representative liquid biopsy applications in hepatocellular carcinoma.

Evidence tier	Clinical application	Representative analyte/assay strategy	Sample source	Disease context	Key representative evidence	Validation status	Regulatory/guideline status	Major limitations
Tier 1—clinically established	No representative Tier 1 liquid‐biopsy application identified	—	—	HCC	Current evidence supports several advanced translational strategies, but no application was assigned to Tier 1 in this evidence framework.	—	No routine Tier 1 liquid‐biopsy use assigned in this table.	—
Tier 2—late‐stage clinical translation	Early detection and prognostic stratification	Plasma ctDNA methylation panel (HepaAiQ‐type HCC‐specific DMR panel)	Plasma cfDNA	HCC across stages; high‐risk liver disease cohorts	A multicenter cohort reported high discrimination in training and validation sets and postoperative prognostic association [167].	Prospective multicenter discovery/validation	Investigational; prospective real‐world surveillance utility still required.	Predominantly Asian/HBV‐enriched cohorts; lower sensitivity in very early disease; downstream management impact not yet established.
Tier 3—early‐stage clinical validation	Early detection	Blood DNA methylation classifiers (e.g., epiLiver/PBMC methylation and plasma methylated‐DNA panels)	PBMC DNA and/or plasma cfDNA	Early‐stage HCC and high‐risk controls	Prospective and phased validation studies support biological and clinical validity of methylation‐based detection [168, 169].	Single‐center prospective or multiphase clinical validation	Investigational	Source biology differs between PBMC and plasma assays; external validation, etiologic diversity, and prospective surveillance performance remain limited.
Tier 3—early‐stage clinical validation	Postoperative MRD/recurrence‐risk stratification	Tumor‐naive ctDNA multiomics integrating copy‐number, fragmentation and AFP	Plasma cfDNA	Resected HCC	A prospective cohort associated postoperative molecular positivity with substantially worse survival [176].	Prospective single‐center clinical validation	Investigational	Small cohort; tumor‐naive sensitivity and optimal postoperative timing are uncertain; no validated MRD‐guided treatment strategy.
Tier 3—early‐stage clinical validation	Molecular profiling, treatment‐response/resistance assessment, and prognosis	Targeted ctDNA mutation profiling (TERT, TP53, CTNNB1, PI3K/MTOR‐pathway and other recurrent alterations)	Plasma cfDNA	Advanced HCC and selected postoperative cohorts	Clinical cohorts show feasibility of mutation detection and associations between selected alterations and resistance/outcomes [166, 172].	Retrospective/prospective observational clinical cohorts	Investigational; no general regulatory claim assigned here	Early‐stage sensitivity is low; panel design and depth strongly affect yield; many recurrent HCC alterations are not directly actionable; CHIP can confound low‐VAF calls.
Tier 3—early‐stage clinical validation	Diagnosis/prognosis/response phenotyping	CTC enumeration and phenotyping, including mesenchymal and PD‐L1‐positive CTCs	Peripheral blood	HCC across stages; systemic‐therapy cohorts	Meta‐analytic and recent clinical cohort evidence supports prognostic associations and potential response phenotyping [179, 182].	Retrospective cohorts and meta‐analyses; early prospective validation	Research‐use/investigational	No standardized CTC platform; low abundance in early disease; subtype findings require multicenter validation.
Tier 3—early‐stage clinical validation	Early detection and prognostic stratification	EV‐associated RNA/protein signatures and circulating miRNA panels (e.g., LINC00853, EV ECG score, serum miRNA panels)	Plasma/serum EVs; serum	Early‐stage and AFP‐negative HCC	Independent clinical cohorts report strong diagnostic signals for selected EV/miRNA assays [187, 192, 195].	Retrospective cohorts with internal/independent validation	Investigational	EV isolation and normalization are heterogeneous; many cohorts are single‐center and HBV‐enriched; prospective screening utility is unproven.
Tier 4—discovery and mechanistic exploration	Mechanistic biomarker and therapeutic‐target exploration	circRNAs/lncRNAs (e.g., circMTO1, circSETD3 and related RNA pathways)	Tumor tissue, cell models, experimental systems; limited circulating correlates	HCC biology and progression	Experimental studies implicate circular/long noncoding RNAs in HCC growth and signaling [196, 197].	Mechanistic/preclinical evidence with limited clinical correlation	Not clinically validated	Predominantly tissue/cell‐model evidence; no validated liquid‐biopsy threshold, prospective clinical utility, or regulatory pathway.

Abbreviations: AFP, alpha‐fetoprotein; cfDNA, cell‐free DNA; CHIP, clonal hematopoiesis of indeterminate potential. Entries are representative rather than exhaustive and are intended to mirror the principal use cases discussed in the corresponding manuscript section; CTC, circulating tumor cell; DMR, differentially methylated region; EV, extracellular vesicle; HCC, hepatocellular carcinoma; MRD, minimal residual disease; PBMC, peripheral blood mononuclear cell; VAF, variant allele fraction.

### 3.3 Colorectal Cancer

#### 3.3.1 Disease Background and Unmet Clinical Needs

CRC is the third most common cancer worldwide and the second leading cause of cancer death. Its incidence and burden are rising globally. CRC typically follows a well‐defined adenoma–carcinoma sequence, progressing over several years from benign or precancerous polyps to invasive and metastatic disease.

Outcomes depend heavily on stage. Five‐year survival exceeds 90% for Stage I CRC but drops to about 72% in locally advanced disease and to roughly 14% in Stage IV CRC [198]. Fewer than half of cases are diagnosed at a localized stage, largely because early disease is often asymptomatic. This prolonged preclinical phase offers a window for early detection that current practice has not fully exploited.

Conventional CRC screening and diagnostic modalities suffer from important limitations. Colonoscopy and tissue biopsy are invasive, costly, require complex patient preparation, and rely on single‐site sampling that fails to capture intratumoral heterogeneity. Imaging‐based approaches, including computed tomographic colonography, expose patients to ionizing radiation and show limited sensitivity for early‐stage lesions. Stool‐based tests such as the fecal immunochemical test (FIT) and multitarget stool DNA assays have improved population screening, but sensitivity remains lower for advanced precancerous lesions than for established CRC, adherence is variable, and positive results require confirmatory colonoscopy [199, 200]. Serum tumor markers such as CEA and CA19‐9 are primarily useful for monitoring advanced disease and lack sensitivity for early detection [201].

These limitations highlight the need for minimally invasive, repeatable biomarkers for early detection, longitudinal monitoring, and treatment guidance in CRC. Liquid biopsy has emerged as a minimally invasive approach, leveraging diverse circulating analytes—ctDNA, CTCs, EVs, TEPs, ncRNAs, and microbiome‐associated signals—to address these gaps [202].

#### 3.3.2 Early Detection and Screening

##### 3.3.2.1 SEPT9 Methylation‐Based Blood Screening

ctDNA, released from apoptotic or necrotic CRC cells, represents one of the most extensively studied liquid biopsy analytes in CRC. Blood‐based CRC detection has increasingly shifted from total cfDNA abundance toward tumor‐associated molecular features, particularly DNA methylation and genome‐wide fragmentation patterns. Total cfDNA concentration lacks sufficient tumor specificity for screening because benign colorectal lesions and other sources of cell turnover can also contribute circulating DNA [203].

SEPT9 promoter hypermethylation is an established example of clinical translation in blood‐based CRC screening [204, 205, 206, 207]. It forms the basis of Epi proColon, an United States Food and Drug Administration‐approved blood‐based screening test with a restricted indication for average‐risk adults aged ≥50 years who have been offered but have not completed recommended CRC screening. Positive results require diagnostic colonoscopy, and the assay is not intended to replace recommended stool‐based or endoscopic screening [208].

##### 3.3.2.2 cfDNA Fragmentomics

Fragmentomics‐based approaches are emerging as noninvasive screening tools. A multicenter study (*n* = 394) reported an AUC of 0.926 for CRC detection, with 91.3% sensitivity, 82.3% specificity, and 94.4% sensitivity for Stage I. FRAGTECT (*n* = 838) achieved a validation AUC of 0.986 (94.9% sensitivity, 98% specificity). These findings support fragmentomics as an emerging blood‐based strategy for CRC detection [209, 210, 211].

##### 3.3.2.3 cfDNA Methylation Panels

To improve diagnostic specificity, ctDNA‐based CRC screening strategies have increasingly focused on tumor‐associated methylation patterns and multimarker panels. Multiple studies have demonstrated that methylation‐based assays outperform mutation‐only approaches in early‐stage disease. For example, cfDNA methylation models targeting panels of 11 or more markers significantly enhance early CRC detection [212], while an MCTA‐Seq‐based panel of 80 methylation markers achieved sensitivities of 62, 81, and 85% for Stage I, II, and III/IV CRC, respectively, with a specificity of 86% [213].

Tumor‐specific methylation markers have also demonstrated high performance across disease stages. Jensen et al. reported that methylation of C9orf50, KCNQ5, and CLIP4 achieved sensitivities of 80, 85, 89, and 88% for Stages I–IV CRC, respectively, with a specificity of 99% [214, 215]. Additional panels such as cg10673833 (89.7% sensitivity, 86.8% specificity, AUC 0.90), SDC2+SEPT9 (CRC sensitivity 88.9%, Stage I 80.0%), and HAND2+GPM6A (AUC 0.921) have demonstrated robust performance [216, 217, 218, 219, 220, 221].

##### 3.3.2.4 Circulating Tumor Cells

CTC detection in CRC has historically been limited by low sensitivity. Early approaches based on CK20 RT‐PCR achieved detection rates of only ∼30%, restricting clinical utility for early screening [222]. Recent technological innovations have substantially improved CTC capture and analysis. The CellMax platform demonstrated a sensitivity of 89% and specificity of 82.1% in distinguishing CRC patients from healthy controls [223]. Similarly, a centrifugal microfluidic FAST system achieved 75% sensitivity and 100% specificity when using a threshold of five CTCs per 7.5 mL of blood [220].

Combinatorial strategies further enhance diagnostic performance. Integration of CTC enumeration with FIT or CEA testing reduces false‐positive rates and compensates for the limited sensitivity of individual assays [224]. Mesenchymal CTCs are associated with aggressive disease and inferior PFS [225], while expression of markers such as PRL‐3, MMP9, TYMS, and RAD23B predicts treatment response and survival outcomes [226, 227].

##### 3.3.2.5 EV and TEP Biomarkers

Multiple EV‐derived biomarkers demonstrate strong diagnostic potential. Plasma exosomal QSOX1 shows high discriminatory performance (AUC 0.904, 85.1% sensitivity) [228], while elevated exosomal GPC1 and CPNE3 levels distinguish CRC patients from controls [229, 230]. Exosomal miRNA panels further enhance early detection and metastatic discrimination, with combined signatures outperforming single markers [231, 232, 233].

TEPs have also been evaluated in CRC. Platelet RNA profiling has differentiated CRC from healthy donors and patients with noncancerous intestinal diseases in clinical cohorts [98, 99]. TEP‐derived TIMP1 mRNA has additionally shown diagnostic value in CRC [97].

#### 3.3.3 Molecular Profiling and Therapy Selection

The diagnostic and prognostic value of ctDNA in CRC derives from its enrichment for tumor‐specific genetic and epigenetic alterations, including recurrent mutations (KRAS, NRAS, BRAF V600E, PIK3CA), copy‐number aberrations, MSI (MSI‐H), tumor mutation burden (TMB), and aberrant DNA methylation (SEPT9, SDC2, SFRP2, BCAT1) [234, 235, 236, 237].

RAS and BRAF V600E characterization is clinically established for treatment selection in metastatic CRC. Plasma ctDNA provides a complementary route to molecular profiling, particularly when tissue is unavailable, inadequate, or unsuitable for repeat sampling. These uses are supported by molecular‐testing guidelines and recommendations for validated plasma ctDNA assays in advanced cancer [129, 238]. BRAF V600E also identifies a clinically relevant poor‐prognosis subgroup and informs BRAF‐targeted treatment selection.

#### 3.3.4 MRD Assessment and Recurrence Prediction

One of the most compelling clinical applications of ctDNA in CRC is the assessment of MRD following curative‐intent surgery. Postoperative ctDNA positivity is associated with a markedly increased risk of recurrence, with studies reporting up to a sevenfold higher recurrence risk at 30 days after surgery compared with ctDNA‐negative patients [239]. Long‐term follow‐up further demonstrates significantly inferior RFS and OS among ctDNA‐positive individuals.

The DYNAMIC‐III trial (*n* = 968) demonstrated that ctDNA‐negative patients achieved 3‐year RFS of 87% compared with 49% for ctDNA‐positive patients. In the GALAXY study, ctDNA MRD positivity was associated with a DFS hazard ratio of 9.69, substantially outperforming CEA (HR 2.13) [240, 241].

Clinical utility has also been tested prospectively. In Stage II colon cancer, the DYNAMIC trial reduced adjuvant chemotherapy use without compromising RFS [242]. In Stage III disease, DYNAMIC‐III reduced treatment intensity among ctDNA‐negative patients but did not meet the prespecified noninferiority margin; treatment escalation in ctDNA‐positive patients did not improve RFS [240]. These findings indicate that the clinical utility of MRD‐guided management is stage‐ and intervention‐specific.

##### 3.3.4.1 EV‐Based MRD Risk Stratification

The CLEAR study evaluated an exosome‐based five‐miRNA signature for postoperative molecular residual‐disease risk stratification in Stage II–III CRC and linked the molecular signature to disease‐free survival [243].

#### 3.3.5 Treatment Response and Resistance Monitoring

In advanced or metastatic CRC, ctDNA enables dynamic monitoring of treatment response and acquired resistance. Baseline cfDNA levels correlate with prognosis in patients receiving immunochemotherapy [244], and postoperative ctDNA detection in patients with resected colorectal liver metastases predicts inferior recurrence‐free and OS [245]. cfDNA fragmentomic features have also been investigated for treatment monitoring and prognostic assessment [209].

Liquid biopsy has further elucidated resistance mechanisms to anti‐EGFR therapies, including re‐emergence of RAS pathway alterations and clonal evolution under treatment pressure [235, 246, 247]. Serial monitoring of RAS/BRAF mutations in ctDNA can detect emerging resistance prior to radiographic progression, providing an earlier molecular signal of clonal evolution.

EVs also actively mediate resistance to chemotherapy. CAF‐derived exosomal lncRNA CCAL activates β‐catenin signaling to promote oxaliplatin resistance, while exosomal Wnt proteins and miR‐196b‐5p contribute to 5‐FU resistance [248, 249]. These findings define additional mechanisms of acquired resistance but remain distinct from clinically validated liquid‐biopsy assays. Engineered EVs delivering therapeutic nucleic acids have further emerged as innovative drug delivery platforms [250].

#### 3.3.6 Emerging Biomarkers: ncRNAs

ncRNAs, including miRNAs, lncRNAs, and circRNAs, have been extensively investigated as CRC biomarkers. Multiple miRNA panels demonstrate diagnostic potential, including miR‐21 (plasma/saliva), miR‐92a‐3p (plasma/serum), miR‐29c/miR‐149 (serum), and miR‐451a (serum).

LncRNAs such as DANCR (prognostic, AUC 0.747), SNHG11 (diagnostic, 4‐lncRNA panel AUC 0.937), and CCAT1 (therapy‐related) have been evaluated in retrospective cohorts. CircRNAs including circ‐CCDC66 (CRC AUC 0.920, 94% sensitivity, 72% specificity; combined with CEA/CA19‐9 → AUC 0.991 for polyp vs. healthy differentiation), circ‐ABCC1, circ‐STIL, and circCAMSAP1 have demonstrated diagnostic and prognostic potential [251, 252, 253, 254, 255].

Mechanistic studies have further elucidated ncRNA‐driven oncogenesis. circ‐CCDC66 knockdown suppresses proliferation/migration/invasion via miRNA sponge mechanisms (DNMT3B/EZH2/MYC/YAP1). circ‐ABCC1 promotes stemness via exosomal Wnt/β‐catenin signaling. SNHG20 sponges miR‐495 to activate STAT3, while CCAT1 is linked to chemoresistance [256, 257, 258].

#### 3.3.7 Evidence Maturity and Translational Status

Liquid biopsy applications in CRC span four levels of evidence maturity (Table 3). Tier 1—clinically established includes two defined clinical contexts. SEPT9 promoter methylation‐based blood testing has regulatory approval for a restricted CRC screening indication in individuals who have not completed recommended screening, while plasma ctDNA genotyping and comprehensive genomic profiling support actionable molecular characterization in selected metastatic CRC settings. Tier 2—late‐stage clinical translation includes postoperative ctDNA MRD assessment and cfDNA fragmentomics. MRD has strong prospective prognostic evidence and has entered biomarker‐guided adjuvant‐treatment trials, whereas fragmentomic classifiers have undergone multicenter validation for detection and longitudinal assessment. Tier 3—early‐stage clinical validation encompasses additional cfDNA methylation panels, CTC‐based assays, EV‐derived protein and miRNA signatures, TEP markers, and circulating ncRNA classifiers evaluated in clinical cohorts. Tier 4—discovery and mechanistic exploration comprises predominantly tissue‐, cell‐, and model‐based circRNA and lncRNA pathways involved in CRC growth, metastasis, and treatment resistance. Overall, CRC illustrates why evidence maturity should be assigned to specific clinical use cases rather than to an analyte as a whole: plasma ctDNA is clinically established for selected molecular‐profiling applications, highly advanced for postoperative MRD, and still investigational for several early‐detection and multianalyte strategies.

**TABLE 3:** Evidence maturity of representative liquid biopsy applications in colorectal cancer.

Evidence tier	Clinical application	Representative analyte/assay strategy	Sample source	Disease context	Key representative evidence	Validation status	Regulatory/guideline status	Major limitations
Tier 1—clinically established	Blood‐based CRC screening in selected adults who have not completed recommended screening	SEPT9 promoter methylation blood test (Epi proColon)	EDTA plasma	Average‐risk adults meeting the labeled indication	Epi proColon is indicated for average‐risk adults aged ≥50 years who have not completed recommended CRC screening; positive results require diagnostic colonoscopy [208].	Clinical validation and regulatory approval for a restricted screening indication	US FDA‐approved for a restricted screening indication; should not be represented as a replacement for recommended stool‐based or endoscopic screening.	Low sensitivity for advanced precancerous lesions; limited positive predictive value in low‐prevalence settings; positive tests require colonoscopy.
Tier 1—clinically established	Actionable molecular profiling in metastatic CRC	Plasma ctDNA genotyping/CGP for actionable alterations, including BRAF V600E and RAS‐pathway status in appropriate contexts	Plasma cfDNA	Metastatic CRC	Guidelines support RAS/BRAF characterization in metastatic CRC; validated plasma ctDNA assays can complement tissue profiling in selected settings [129, 238].	Guideline‐supported molecular stratification and clinical validation of plasma ctDNA profiling	Clinically established in selected molecular‐profiling settings; use is biomarker‐, assay‐, and context‐specific.	Low ctDNA shedding can yield false negatives; CHIP and low‐VAF artifacts can confound some variants; negative or discordant results may require tissue confirmation.
Tier 2—late‐stage clinical translation	MRD assessment, recurrence prediction, and adjuvant‐risk stratification	Tumor‐informed and tumor‐naive ctDNA MRD assays	Plasma	Resected Stage II–III colon/CRC	DYNAMIC‐III and GALAXY‐type studies show strong prognostic stratification and feasibility of ctDNA‐guided management, while definitive de‐escalation utility remains under evaluation [240, 241].	Prospective multicenter trials and large prospective cohorts	MRD is clinically prognostic, but routine treatment escalation/de‐escalation based solely on ctDNA is not yet established.	Tumor‐informed assays require tissue and turnaround time; tumor‐naive sensitivity varies; clinical‐utility endpoints and action thresholds remain unresolved.
Tier 2—late‐stage clinical translation	Early detection and treatment monitoring	cfDNA fragmentomics (CNV, fragmentation patterns and multifeature classifiers)	Plasma cfDNA	CRC across stages; high‐risk cohorts; metastatic monitoring	Multicenter studies report high discrimination and treatment‐response/prognostic signals [209 ^−^ 211].	Prospective multicenter cohorts with independent validation	Investigational	Average‐risk screening utility is not established; advanced adenoma sensitivity is lower than CRC sensitivity; computational pipelines and thresholds are not standardized.
Tier 3—early‐stage clinical validation	Early detection, recurrence monitoring and prognosis	cfDNA methylation panels and CTC‐based assays	Plasma cfDNA; peripheral blood CTCs	CRC across stages and postoperative follow‐up	Methylated BCAT1/IKZF1 and broader methylation panels, together with prospective CTC studies, support early clinical validity [216, 217, 219, 220].	Prospective/observational clinical cohorts	Investigational; commercial LDTs exist for selected methylation assays.	Cutoffs and platforms vary; sensitivity for advanced adenomas is limited; CTC rarity and enrichment heterogeneity constrain reproducibility.
Tier 3—early‐stage clinical validation	Early diagnosis and molecular residual‐disease risk assessment	EV protein signatures, TEP mRNA and exosomal miRNA panels	Serum/plasma EVs; platelets	CRC diagnosis and postoperative Stage II–III disease	QSOX1, platelet TIMP1 and exosomal miRNA panels show diagnostic signals; the CLEAR study links an exosome‐based signature to postoperative risk [97, 228, 243].	Case–control, observational, and multicenter retrospective validation	Investigational	Small cohorts, assay‐specific isolation/QC, limited external prospective validation, and no established management algorithm.
Tier 3—early‐stage clinical validation	Exploratory diagnosis and prognosis	Circulating ncRNA signatures (plasma circRNAs and lncRNAs such as circ‐CCDC66 and SNHG11)	Plasma/serum	CRC and colorectal neoplasia	Single‐center clinical studies report diagnostic and prognostic associations [251, 253].	Retrospective single‐center clinical validation	Investigational	Small samples, normalization heterogeneity, overfitting risk, and limited independent validation.
Tier 4—discovery and mechanistic exploration	Mechanistic ncRNA biology	circRNA/lncRNA pathways in CRC (e.g., circ‐CCDC66 and related networks)	Tumor tissue, cell lines, and xenograft models	CRC pathogenesis and therapeutic‐target exploration	Mechanistic studies support roles in proliferation, stemness and metastasis [257].	Preclinical/mechanistic	Not clinically validated	Not a validated liquid‐biopsy application; clinical assay development and prospective utility evidence are lacking.

Abbreviations: CDx, companion diagnostic; cfDNA, cell‐free DNA; CGP, comprehensive genomic profiling; CHIP, clonal hematopoiesis of indeterminate potential. Entries are representative rather than exhaustive and are intended to mirror the principal use cases discussed in the corresponding manuscript section; CRC, colorectal cancer; CTC, circulating tumor cell; ctDNA, circulating tumor DNA; EV, extracellular vesicle; LDT, laboratory‐developed test; mCRC, metastatic CRC; MRD, minimal residual disease; TEP, tumor‐educated platelet.

### 3.4 Breast Cancer

#### 3.4.1 Disease Background and Unmet Clinical Needs

BC is the most prevalent malignancy among women worldwide and remains a major contributor to global cancer‐related morbidity and mortality. Each year, approximately 2.3 million new cases are diagnosed, accounting for nearly one quarter of all cancers in women and ∼14% of female cancer‐related deaths [259, 260]. A defining feature of BC is its marked biological heterogeneity, encompassing diverse molecular subtypes, evolutionary trajectories, and therapeutic vulnerabilities, which collectively contribute to disease recurrence and treatment resistance.

Although population‐based screening programs—most notably mammography—enable detection of early‐stage disease in a substantial proportion of patients, long‐term outcomes remain suboptimal. Approximately 20% of patients diagnosed with apparently localized, early‐stage BC ultimately develop incurable metastatic relapse, suggesting that occult dissemination may already be present at diagnosis [261, 262]. Given the strong association between advanced‐stage disease and poor prognosis, early detection, accurate risk stratification, and timely therapeutic intervention remain central goals in BC management.

Current diagnostic and monitoring strategies face important limitations. Mammography, while effective for many patients, shows reduced sensitivity in dense breast tissue and certain molecular subtypes, and cannot capture molecular heterogeneity [263, 264]. Tissue biopsy, the cornerstone for molecular classification and therapeutic decision‐making, is invasive, subject to sampling bias, and poorly suited for longitudinal monitoring of tumor evolution [265, 266]. These constraints have driven increasing interest in liquid biopsy as a minimally invasive, repeatable approach to capture tumor dynamics across the disease course.

#### 3.4.2 Early Detection and Screening

##### 3.4.2.1 ctDNA‐Based Early Detection

ctDNA, released from apoptotic and necrotic BC cells, has been extensively investigated for early BC detection. Plasma ctDNA levels are higher in BC patients than in individuals with benign breast disease or healthy controls, supporting its diagnostic potential [267, 268]. Early studies using DNA integrity indices further demonstrated that ctDNA fragmentation patterns correlate with tumor presence and TNM stage [269].

ctDNA enables detection of BC‐specific genetic and epigenetic alterations, including somatic mutations (PIK3CA, TP53, ESR1), LOH, MSI, and promoter methylation. Notably, PIK3CA mutations—present in approximately 30% of BCs—can be detected in plasma ctDNA with high sensitivity and specificity even in early‐stage disease [270]. Using targeted error‐corrected sequencing, ctDNA has been detected in up to 67% of Stage I BC patients, although detection rates decline with decreasing tumor burden, reflecting ultra‐low mutant allele fractions in localized disease [271, 272].

Methylation‐based ctDNA assays further enhance early detection. Bisulfite sequencing studies have identified differential promoter methylation of genes such as EGFR and PPM1E in BC patients, underscoring the added value of epigenetic profiling beyond mutation‐only approaches [267]. Methylation panels such as SPAG6, PER1, NKX2‐6, and ITIH5 (SNiPER panel) have been explored for early detection [273].

##### 3.4.2.2 cfDNA Multimodal Analysis

cfDNA multimodal approaches integrating methylation, fragmentomics, copy‐number alterations, and end motifs have demonstrated robust early detection performance. The SPOT‐MAS assay (*n* = 2288) achieved early‐stage sensitivities of 73.9–62.3% across stages. The TuFEst assay (*n* = 792) demonstrated 95% sensitivity for early detection at 78.3% specificity. Van et al. (*n* = 515) reported an AUC of 0.90 with 93.6% specificity. These studies support the value of integrating complementary cfDNA features for early BC detection, although population‐level screening utility has not yet been established [274, 275, 276, 277].

##### 3.4.2.3 EV‐Derived miRNA Panels

EV‐derived miRNAs have shown promise for risk stratification of indeterminate breast lesions. A 4‐miRNA panel (miR‐340‐5p/miR‐15b‐5p from HER2^+^ EVs and miR‐598‐3p/miR‐126‐3p from CD24^+^ EVs), detected using the TENPO platform in BI‐RADS 4 lesions (86 benign, 27 malignant), achieved an AUC of 0.95 with 87% sensitivity and 91% specificity. miR‐340‐5p alone reached an AUC of 0.87, and qPCR validation confirmed correlation (Pearson's *R* = 0.71) [278]. This panel was developed to improve benign‐versus‐malignant risk stratification in patients with BI‐RADS 4 lesions.

##### 3.4.2.4 ncRNA and Alternative Biomarkers

Circulating ncRNAs, including miRNAs, lncRNAs, and circRNAs, constitute an important component of the BC liquid biopsy landscape. Serum miRNA panels have demonstrated diagnostic potential in early‐stage BC, while circulating miRNA profiles have also been associated with primary and metastatic disease [279, 280].

Additional circulating biomarkers—including salivary mRNAs and proteins (S100A8, GRIK1, GRM1, H6PD, IGF2BP1, CSTA, MDM4, TPT1 mRNA; CA6, CA125, sFas proteins), as well as volatile organic compounds (VOCs) detected in urine (2‐propanol, 2‐butanone)—have demonstrated promising diagnostic performance for early BC detection, representing candidate noninvasive approaches for BC detection [281, 282, 283].

#### 3.4.3 Molecular Profiling and Therapy Selection

BC management relies on comprehensive molecular profiling to guide therapy selection [284, 285, 286]. Tissue‐based biomarkers—including HER2 IHC/ISH, ER/PR IHC, and PD‐L1 CPS—remain the gold standard for molecular classification and are well‐established for guiding therapy selection. However, tissue biopsy is invasive, subject to sampling bias, and poorly suited for longitudinal monitoring. Liquid biopsy, particularly ctDNA, has emerged as a complementary approach for detecting actionable biomarkers when tissue is limited or for serial monitoring, capturing spatial and temporal heterogeneity, and identifying resistance mechanisms.

Among liquid‐biopsy applications, plasma ctDNA testing for PIK3CA and ESR1 mutations has entered defined clinical use in HR‐positive/HER2‐negative advanced or metastatic BC, where mutation status can inform treatment selection. Testing remains assay‐ and indication‐specific, and a negative plasma result may require tissue confirmation. These applications remain complementary to tissue‐based HER2, ER/PR, and PD‐L1 assessment [129, 286].

Beyond these established biomarker–therapy settings, other ctDNA‐detected alterations, including HER2 amplification, CDK4/6‐related alterations, and FGFR alterations, have been associated with treatment resistance or prognosis in clinical studies, supporting the value of serial molecular profiling but remaining distinct from regulatory companion‐diagnostic use.

#### 3.4.4 MRD Assessment and Recurrence Prediction

##### 3.4.4.1 ctDNA MRD

ctDNA MRD assessment has demonstrated strong prognostic value for recurrence prediction in early‐stage BC. Tumor‐informed bespoke panels using WES‐identified patient‐specific variants and tracked via multiplex PCR, dPCR, or NGS have demonstrated robust prognostic performance [287, 288, 289, 290, 291].

In the PREDICT study, ctDNA positivity was associated with a recurrence HR of 128, while ctDNA‐negative patients achieved 5‐year invasive disease‐free survival of 94%. The ALIENOR study reported a lead time of 13.9 months for ctDNA‐detected recurrence before clinical or radiographic detection. In the c‐TRAK TN trial, ctDNA was detected in 27.3% of high‐risk TNBC patients during 12 months of surveillance. Importantly, c‐TRAK TN also highlighted the gap between molecular detection and effective intervention: 72% of ctDNA‐positive patients allocated to intervention already had metastatic disease on staging, and none of the five patients who initiated pembrolizumab achieved sustained ctDNA clearance [288]. Thus, current BC MRD evidence strongly supports recurrence‐risk stratification, whereas the clinical benefit of MRD‐triggered systemic intervention remains unestablished.

##### 3.4.4.2 CTC‐Based Recurrence Risk

CTCs are detectable throughout BC progression and play a pivotal role in metastatic dissemination. In advanced disease, CTC enumeration using the CellSearch system has demonstrated robust prognostic value, with counts ≥5 CTCs/7.5 mL blood consistently associated with shorter progression‐free and OS [292].

In early‐stage BC, however, the extreme rarity of CTCs poses significant analytical challenges. Only a minority of patients exhibit detectable CTCs prior to neoadjuvant therapy, limiting sensitivity for early diagnosis and risk prediction [293]. Recent technological advances have begun to address these limitations. Size‐ and deformability‐based enrichment platforms (e.g., Parsortix), microfluidic devices (CTC‐iChip), and label‐free nanotube‐based capture systems enable broader phenotypic coverage, including EpCAM‐low and EMT‐like CTCs [294, 295, 296, 297].

#### 3.4.5 Treatment Response and Resistance Monitoring

Subtype‐specific ctDNA alterations play a central role in resistance monitoring. ESR1 mutations in ctDNA are strongly associated with resistance to aromatase inhibitors in HR+ metastatic BC. In the PADA‐1 trial, early detection of rising ESR1 mutation levels enabled timely switching of endocrine therapy prior to clinical progression and was associated with improved PFS in the trial population [298, 299]. PADA‐1 therefore provides interventional evidence that ctDNA monitoring can alter treatment management before clinical progression in a defined biomarker–therapy setting [298, 299].

TP53 mutations detected in plasma ctDNA correlate with inferior PFS across treatment modalities [300]. Large clinical trials, including MONALEESA‐ASIA, further confirmed that baseline ctDNA burden and specific alterations (PIK3CA, TP53) predict shorter progression‐free and OS in patients receiving CDK4/6 inhibitor‐based therapy [301].

HER2 amplification detected in ctDNA has been associated with trastuzumab resistance and disease progression. CDK4/6 amplification in ctDNA has been associated with CDK4/6 inhibitor resistance and poor prognosis [302]. FGFR1 amplification has been associated with resistance to endocrine therapy and CDK4/6 inhibition in HR‐positive metastatic BC [303]. These resistance‐associated alterations remain distinct from the interventional evidence supporting ESR1‐guided treatment switching in PADA‐1.

#### 3.4.6 Prognostic Stratification

##### 3.4.6.1 CTC‐Based Prognosis

In advanced BC, CTC enumeration using the CellSearch system has demonstrated robust prognostic value, with counts ≥5 CTCs/7.5 mL blood consistently associated with shorter PFS and OS [292]. Beyond enumeration, molecular and phenotypic profiling of CTCs has shown prognostic and treatment‐response associations in clinical studies. Detection of ESR1 mutations, CK‐19 mRNA‐positive CTCs, and mesenchymal or immune‐evasive phenotypes (e.g., PD‐L1^+^ or CD47^+^ CTCs) correlates with treatment resistance, metastatic progression, and inferior survival outcomes [304, 305, 306]. Emerging metabolic and transcriptomic signatures further refine risk stratification [307].

##### 3.4.6.2 ctDNA‐Based Prognosis

In early‐stage disease, detection of PIK3CA or TP53 mutations in ctDNA identifies patients at higher risk of recurrence [300, 308]. These data support recurrence‐risk stratification but do not establish a role for ctDNA‐guided adjuvant treatment intensification. In advanced and metastatic BC, ctDNA profiling informs targeted therapy selection and prognosis [309]. The MONARCH 3 substudy demonstrated that abemaciclib benefit was independent of baseline alterations (HR 0.49) [302].

##### 3.4.6.3 TEP‐Based Prognosis

TEPs acquire tumor‐derived RNA and protein signatures through direct and indirect interactions with BC cells, reflecting systemic tumor–host crosstalk. TEP‐associated markers such as PD‐L1 and 11‐dehydro‐thromboxane B2 correlate with metastatic burden, treatment response, and survival outcomes [310, 311].

#### 3.4.7 Emerging Biomarkers and Multianalyte Integration

##### 3.4.7.1 Noncoding RNAs

miRNAs are the most extensively characterized ncRNAs in BC. Multi‐miRNA panels have achieved high sensitivity for BC detection, including in situ carcinoma, and enable discrimination between localized and metastatic disease [279, 280]. Several miRNA signatures also predict pathological complete response to neoadjuvant therapy and stratify relapse risk. miR‐23b, miR‐200c, and miR‐190 predict relapse in localized BC [312]. miR‐148a‐3p and miR‐374a‐5p predict pCR after trastuzumab‐based neoadjuvant therapy [313]. miR‐503 predicts response to taxane and anthracycline neoadjuvant chemotherapy [314].

lncRNAs and circRNAs are under investigation as circulating biomarkers, particularly when packaged within EVs. Exosomal lncRNA H19 has demonstrated diagnostic potential (AUC 0.870, 87.0% sensitivity, 70.6% specificity) and has shown associations with clinicopathological features [315]. Plasma circHIPK3 has shown diagnostic potential (AUC 0.809) [316].

##### 3.4.7.2 Extracellular Vesicles

EVs protect RNA cargo from degradation and reflect tumor–microenvironment interactions. EV‐derived long RNA and miRNA profiles have shown diagnostic or treatment‐response associations in BC [317, 318]. Exosomal HSP70 has also been explored as a circulating marker during clinical follow‐up in patients with cancer [319]. Beyond their diagnostic role, EVs actively mediate tumor progression and therapy resistance. EV‐associated proteins and regulatory RNAs modulate immune evasion, metastatic niche formation, and drug response, highlighting EVs as both biomarkers and therapeutic targets [318, 319].

##### 3.4.7.3 TEPs and Alternative Biomarkers

Platelet RNA profiling has achieved high accuracy in BC detection and subtype discrimination, including identification of HER2‐amplified, PIK3CA‐mutant, and triple‐negative disease [310, 311]. TEP‐PSGL1 drives stemness and EMT via the WNT–β‐catenin–VEGF–VEGFR2 axis in preclinical models [320].

VOCs detected in urine (2‐propanol and 2‐butanone) have demonstrated an AUC of 0.944 (93.3% sensitivity, 83.3% specificity) for early BC detection [283].

#### 3.4.8 Evidence Maturity and Translational Status

Liquid biopsy evidence in BC spans four evidence tiers. Tier 1—clinically established is represented by plasma ctDNA testing for PIK3CA and ESR1 mutations in defined biomarker–therapy settings in HR‐positive/HER2‐negative advanced or metastatic BC, supported by guideline‐based molecular testing in defined clinical settings. Tissue‐based HER2, ER/PR, and PD‐L1 testing remains outside this liquid‐biopsy tier. Tier 2—late‐stage clinical translation includes tumor‐informed ctDNA MRD assessment after curative‐intent treatment and multimodal cfDNA approaches for early detection. Prospective studies, including PREDICT, ALIENOR, and c‐TRAK TN, demonstrate strong prognostic value for MRD, while multimodal cfDNA studies such as SPOT‐MAS and TuFEst support advanced clinical validation for early detection; neither use case is routinely implemented. Tier 3—early‐stage clinical validation encompasses EV‐derived miRNA panels for breast‐lesion risk stratification, methylation‐based and other ctDNA classifiers, CTC molecular phenotyping, and circulating ncRNA and TEP signatures evaluated in clinical cohorts. Tier 4—discovery and mechanistic exploration comprises exploratory EV‐, circRNA‐, and TEP‐associated biology, including exosomal lncRNA H19, circulating circHIPK3, and TEP signaling, supported primarily by small clinical cohorts and mechanistic or preclinical studies. Overall, BC currently shows the clearest clinically established liquid‐biopsy role in defined therapy‐selection settings, whereas ctDNA MRD and multimodal early detection represent the leading translational frontiers and EV‐, ncRNA‐, CTC‐, and TEP‐based approaches remain at earlier stages of clinical development (Table 4).

**TABLE 4:** Evidence maturity of representative liquid biopsy applications in breast cancer.

Evidence tier	Clinical application	Representative analyte/assay strategy	Sample source	Disease context	Key representative evidence	Validation status	Regulatory/guideline status	Major limitations
Tier 1—clinically established	Molecular profiling and therapy selection	Plasma ctDNA testing for PIK3CA and ESR1 mutations in defined biomarker–therapy settings	Plasma	HR‐positive/HER2‐negative advanced or metastatic breast cancer in defined treatment settings	Guideline‐supported plasma ctDNA testing can inform treatment selection in defined PIK3CA‐ and ESR1‐mutated advanced breast‐cancer settings [129, 286].	Therapeutic evidence and guideline‐supported, assay‐specific molecular testing	Clinically established for defined biomarker–therapy pairs. Clinical use is assay‐ and indication‐specific. Tissue HER2 IHC/ISH, ER/PR and PD‐L1 testing are reference tissue assays and are intentionally excluded from this liquid‐biopsy tier.	Plasma‐tissue discordance and low shedding can produce false negatives; clinical interpretation is assay and therapy specific.
Tier 2—late‐stage clinical translation	Posttreatment MRD and molecular relapse detection	Tumor‐informed bespoke ctDNA MRD assays	Plasma; tumor tissue for assay design	Stage II–III/high‐risk early breast cancer after neoadjuvant or adjuvant therapy	ALIENOR, c‐TRAK TN and longitudinal ctDNA studies show substantial prognostic value and molecular lead time [287, 288, 289].	Prospective multicenter cohorts and interventional trial experience	Investigational for routine MRD‐directed escalation/de‐escalation	High‐risk patients may already have radiographically detectable disease at first ctDNA positivity; brain/low‐shedding disease may be missed; action thresholds are not standardized.
Tier 2—late‐stage clinical translation	Early detection/benign‐versus‐malignant discrimination	Multimodal cfDNA methylation and fragmentomics	Plasma cfDNA	Early‐stage breast cancer, benign breast lesions, and healthy controls	Recent large clinical cohorts show encouraging discrimination and molecular subtyping with multimodal cfDNA features [274, 275, 277].	Multicenter or independently validated clinical cohorts	Investigational; not established for population screening	Most cohorts are case–control or enriched clinical cohorts; population prevalence, downstream work‐up and clinical utility require prospective evaluation.
Tier 3—early‐stage clinical validation	BI‐RADS 4 lesion risk stratification	EV‐derived 4‐miRNA panel from HER2‐positive/CD24‐positive EV subpopulations	Plasma EVs	Indeterminate BI‐RADS 4 breast lesions	A prospective single‐center study reported high discrimination for benign versus malignant lesions [278].	Prospective single‐center validation	Investigational	Small malignant cohort, no independent external validation, EV subpopulation isolation complexity and indication‐specific applicability.
Tier 3—early‐stage clinical validation	Treatment‐response and resistance monitoring; broader molecular profiling	Broader ctDNA panels, methylation classifiers, and CTC molecular profiles	Plasma cfDNA; peripheral blood CTCs	Advanced disease and early‐detection cohorts	Clinical cohort studies support associations of acquired ctDNA alterations and methylation/CTC signatures with response, resistance or diagnosis [273, 302, 321].	Retrospective to early prospective clinical validation	Investigational outside defined Tier 1 CDx indications	Heterogeneous assays and endpoints; many associations are prognostic or correlative rather than management‐changing.
Tier 4—discovery and mechanistic exploration	Exploratory EV/circRNA/TEP biology	Exosomal lncRNA H19, circulating circHIPK3, and tumor‐educated platelet signaling	Serum/plasma; platelets; experimental models	Breast cancer diagnosis, progression, and stemness biology	Exploratory human studies and mechanistic work suggest diagnostic or biological relevance [315, 316, 320].	Small clinical cohorts and mechanistic/preclinical studies	Not clinically validated	Limited external validation, uncertain specificity, and no established clinical decision pathway.

Abbreviations: BI‐RADS, Breast Imaging Reporting and Data System. Entries are representative rather than exhaustive and are intended to mirror the principal use cases discussed in the corresponding manuscript section; CDx, companion diagnostic; cfDNA, cell‐free DNA; CTC, circulating tumor cell; ctDNA, circulating tumor DNA; EV, extracellular vesicle; HER2, human epidermal growth factor receptor 2; HR, hormone receptor; MRD, minimal residual disease; TEP, tumor‐educated platelet.

### 3.5 Gastric Cancer

#### 3.5.1 Disease Background and Unmet Clinical Needs

Gastric cancer (GC) remains one of the most prevalent malignancies worldwide and is the third leading cause of cancer‐related mortality, with over one million new cases diagnosed annually [115]. Despite a global decline in incidence, GC continues to exhibit marked geographic heterogeneity, with substantially higher prevalence in East Asia (particularly Japan and Korea) compared with Western countries. Distinct differences in histological subtypes, molecular features, prognosis, and therapeutic response further distinguish Asian and Western GC populations [322].

Although multimodal treatment strategies—including surgery, chemotherapy, radiotherapy, targeted therapy, and immunotherapy—have advanced considerably, the overall 5‐year survival rate of GC remains unsatisfactory. This is largely attributable to delayed diagnosis: approximately 80% of GC patients are asymptomatic in early stages and are diagnosed only after progression to advanced or metastatic disease, when therapeutic options are limited and prognosis is poor [323, 324]. The prolonged asymptomatic phase between tumor initiation and clinical presentation represents a critical barrier to early detection and effective intervention.

Current diagnostic strategies face substantial limitations. Current diagnostic assessment relies on endoscopy with tissue biopsy, which is invasive and provides a site‐specific sample that may incompletely capture spatial tumor heterogeneity [325, 326]. Serum biomarkers (e.g., CEA, CA19‐9, CA72‐4, CA125, pepsinogen) lack GC specificity and sensitivity, leading to missed diagnoses or false positives [327]. Imaging modalities cannot support real‐time molecular monitoring and expose patients to cumulative radiation risk [328]. Moreover, the cost effectiveness of population‐wide endoscopic screening remains suboptimal in Western countries [323].

Liquid biopsy has emerged as a minimally invasive tool to address these unmet needs in GC—early detection, prognostic stratification, treatment monitoring, resistance assessment, and recurrence surveillance [329, 330].

#### 3.5.2 Early Detection and Screening

##### 3.5.2.1 cfDNA/ctDNA‐Based Early Detection

Circulating cfDNA and ctDNA, primarily released from apoptotic or necrotic GC cells, represent the most extensively investigated liquid biopsy analytes in GC.

Multiple studies have demonstrated that plasma cfDNA/ctDNA levels are significantly elevated in GC patients compared with healthy individuals or patients with benign gastric diseases. Kim et al. reported a cfDNA‐based diagnostic model achieving an AUC of 0.991 in a retrospective case–control setting, while Qian et al. reported that cfDNA concentration showed improved performance compared with conventional serum markers in distinguishing early GC in their cohort [331]. cfDNA/ctDNA levels increase progressively with tumor stage and correlate closely with TNM classification [332, 333].

Epigenetic profiling further enhances early detection. Methylation‐based assays such as MCTA‐Seq and EpiPanGI Dx identify GC‐specific CpG signatures with high specificity, while hypermethylation of tumor suppressor genes (XAF1, RASSF1A, SOX17) is frequently detected in GC‐derived cfDNA but absent in controls [334, 335, 336]. Methylation panels including XAF1 (sensitivity 69.8%), DOCK10/CABIN1/KCNQ5, and RPRML have demonstrated diagnostic potential [336, 337].

##### 3.5.2.2 EV and ncRNA‐Based Early Detection

EVs enhance biomarker stability and biological relevance by encapsulating ncRNAs and proteins. Exosomal 4‐miRNA panel (miR‐34b/miR‐130a/miR‐375/miR‐627) demonstrated an AUC of 0.86 in discovery and 0.83 in validation for early GC detection, with a risk model achieving AUC 0.94 [338]. FR^+^ (folate receptor‐positive) CTCs detected by qPCR have also shown diagnostic associations in clinical cohorts [339, 340].

ncRNAs—including miRNAs, lncRNAs, circRNAs, and tsRNAs—constitute a versatile class of GC liquid biopsy biomarkers. Multiple miRNA panels demonstrate strong diagnostic performance, with miR‐21, miR‐106, miR‐421, and miR‐223 among the most consistently validated [341]. CircRNAs (e.g., circPTPN22) and tsRNAs further provide robust diagnostic and prognostic signals, often reflecting tumor stage, surgical response, and survival risk [342, 343, 344]. Several multimarker EV panels have shown higher diagnostic performance than individual markers in reported cohorts [345].

##### 3.5.2.3 Nonblood Biofluids

Nonblood biofluids—including gastric juice, urine, saliva, stool, and peritoneal fluid—have also been explored as alternative liquid‐biopsy sources. Their proximity to tumor tissue enables higher sensitivity for certain analytes [346, 347, 348, 349]. Gastric juice BARHL2 methylation showed an AUC of 0.923 in the discovery cohort, suggesting potential for early GC detection; independent validation in larger prospective cohorts is needed [347]. Urinary miR‐6807‐5p, miR‐6856‐5p, and miR‐23‐5p have shown potential for early detection and therapy response monitoring (reduced after surgery) [350]. Gastric juice miR‐133a and lncRNA‐AA174084 also demonstrate discriminatory value [351]. Peritoneal exosomal miRNAs (miR‐21‐5p, miR‐92a‐3p, miR‐223‐3p, miR‐342‐3p) indicate peritoneal involvement [352].

#### 3.5.3 Molecular Profiling and Therapy Selection

At the molecular level, ctDNA harbors GC‐specific genetic and epigenetic alterations. Targeted NGS has revealed recurrent mutations (TP53, PIK3CA), copy‐number alterations (HER2, FGFR2 amplification), and MSI. Notably, cfDNA‐based MSI detection achieved >98% overall accuracy, supporting the feasibility of plasma‐based MSI assessment [353, 354].

The VIKTORY umbrella trial (*n* = 772) provided prospective evidence for molecularly matched treatment in advanced GC, while ctDNA analyses in this setting supported the feasibility of plasma‐based molecular profiling. Sequencing was successful in 715 of 772 patients (92.6%), and savolitinib achieved a 50% objective response rate in the MET‐amplified subgroup [355].

ctDNA profiling has identified recurrent alterations in TP53, PIK3CA, ERBB2, KRAS, MET, and FGFR2 in advanced GC, supporting plasma‐based molecular characterization [356, 357]. Plasma ERBB2 and FGFR2 amplification analyses have also been investigated for treatment stratification and resistance monitoring in HER2‐ and FGFR‐directed settings [358, 359].

#### 3.5.4 MRD Assessment and Recurrence Prediction

##### 3.5.4.1 ctDNA MRD

ctDNA MRD assessment has emerged as a powerful tool for recurrence prediction in GC. Tumor‐informed bespoke panels using WES‐identified patient‐specific variants (≥2 variants = MRD+) have demonstrated robust prognostic performance.

In the PLAGAST study, ctDNA positivity was associated with a hazard ratio of 12.94 for RFS and 14.54 for OS [360]. The EXODOX study reported a hazard ratio of 15.8 for ctDNA‐positive patients at baseline (P0) [361]. In the MRD‐GATE study, 1‐year DFS was 57.1% for ctDNA‐positive versus 94.1% for ctDNA‐negative patients (HR 9.66). A meta‐analysis of postoperative ctDNA MRD in GC (*n* = 520) reported a pooled HR of 10.00 for recurrence [362]. Together, these studies establish postoperative ctDNA as a strong recurrence‐risk marker in GC, but they do not yet show that changing adjuvant treatment on the basis of MRD status improves outcomes.

##### 3.5.4.2 CTC and EV‐Based Recurrence Prediction

CTC‐WBC clusters have been associated with poorer OS after radical gastrectomy (HR 2.553) [363]. Peritoneal exosomal miRNAs (miR‐21‐5p, miR‐92a‐3p, miR‐223‐3p, miR‐342‐3p) indicate peritoneal involvement and correlate with peritoneal cancer index (PCI) and recurrence [364].

#### 3.5.5 Treatment Response and Resistance Monitoring

In advanced GC, ctDNA enables real‐time assessment of therapeutic resistance. Alterations such as TGFBR2, RHOA, and PREX2 mutations have been associated with resistance to PD‐1 blockade, while CCNE1 amplification and concurrent MET amplification limit responses to HER2‐ and FGFR2‐targeted therapies, respectively [359, 365, 366].

Dynamic ctDNA level changes further reflect treatment response, with rising levels indicating disease progression [367, 368]. MET amplification detected in ctDNA has been associated with MET inhibitor resistance, particularly in the presence of concurrent KRAS alterations or HER2 amplification [356].

#### 3.5.6 Prognostic Stratification

##### 3.5.6.1 ctDNA‐Based Prognosis

Detectable ctDNA after surgery strongly predicts recurrence, often preceding radiologic detection by several months [332, 369]. Meta‐analyses consistently show that ctDNA positivity is associated with significantly shorter OS, DFS, and PFS [370]. Methylation markers such as XAF1 (HR 5.71) and LINE‐1 hypomethylation are associated with poor prognosis and shorter OS [336, 337]. SOX17, RASSF1A, and Wif‐1 methylation are associated with decreased PFS and OS [337]. TIMP‐3 methylation correlates with peritoneal metastasis and shorter DFS [371].

##### 3.5.6.2 CTC‐Based Prognosis

In advanced GC, CTC counts correlate strongly with metastatic burden and survival outcomes [372, 373, 374]. HER2‐positive CTCs may reveal HER2‐positive circulating tumor clones not captured by a single tissue biopsy, providing complementary molecular information. Chromosomal aneuploidy, PIK3CA mutations, and stem‐like or immune‐evasive phenotypes (e.g., CD44^+^ and PD‐L1^+^ CTCs) correlate with poor outcomes [375, 376, 377, 378]. CD44^+^ CTCs (sensitivity 92.3%) are correlated with distant metastasis and shorter OS [379]. TWIST^+^ CTCs (>2.5 per 7.5 mL blood) are associated with shorter OS [374].

##### 3.5.6.3 ncRNA and EV‐Based Prognosis

Exosomal lncRNA H19 (AUC 0.849) is associated with TNM stage and prognosis [365]. Peritoneal exosomal miRNAs correlate with PCI and recurrence [364]. circPTPN22 has demonstrated diagnostic and prognostic value [342].

#### 3.5.7 Emerging Biomarkers and Multianalyte Integration

##### 3.5.7.1 ncRNA Mechanisms

circPTPN22 inhibits autophagy via the RUNX1/FUS/ELAVL1 axis, demonstrating both diagnostic and mechanistic relevance [354]. tiRNA‐Val‐CAC‐001 suppresses metastasis via LRP6/Wnt/β‐catenin signaling [341]. Exosomal Linc‐ROR (AUC 0.861) regulates the miR‐145‐5p/CARMIL1/ERK‐mTOR pathway [380]. sEV circ6718 (AUC 0.9–0.96) modulates miR‐561‐3p/SAAL1/PRRX1/TGFβ1 to promote CAF differentiation [381]. Peritoneal exosomal miRNAs (miR‐21‐5p, miR‐92a‐3p, miR‐223‐3p, miR‐342‐3p elevation; miR‐29 family downregulation) correlate with peritoneal metastasis and recurrence [364].

##### 3.5.7.2 Integrative Strategies

Recent studies have applied ensemble and machine‐learning models to integrate multidimensional cfDNA features for early gastric‐cancer detection and molecular subtyping [382, 383].

#### 3.5.8 Evidence Maturity and Translational Status

Liquid biopsy applications in GC span several levels of evidence maturity (Table 5), with no representative Tier 1—clinically established application identified under the framework used in this review. Tier 2—late‐stage clinical translation is represented by postoperative ctDNA MRD and recurrence‐risk stratification. Prospective and longitudinal studies consistently associate postoperative ctDNA positivity with a markedly increased risk of recurrence, making MRD the most advanced liquid‐biopsy use case in GC. Evidence that MRD‐directed treatment improves outcomes, however, has not yet been established. Tier 3—early‐stage clinical validation includes exosomal miRNA panels and FR‐positive CTCs for early detection or perioperative risk assessment; ctDNA multigene profiling, ctDNA methylation, and EV‐DNA methylation for molecular or diagnostic stratification; and additional EV‐, CTC‐, gastric‐juice‐, and ncRNA‐based signatures evaluated in clinical cohorts. Tier 4—discovery and mechanistic exploration comprises predominantly EV‐ and ncRNA‐associated mechanisms, including Linc‐ROR, circPTPN22, sEV‐circ6718, and related pathways involved in tumor progression and stromal remodeling. Overall, postoperative ctDNA MRD currently has the strongest translational evidence in GC, whereas molecular profiling, early‐detection, CTC‐, EV‐, and ncRNA‐based applications remain at earlier stages of clinical development.

**TABLE 5:** Evidence maturity of representative liquid biopsy applications in gastric cancer.

Evidence tier	Clinical application	Representative analyte/assay strategy	Sample source	Disease context	Key representative evidence	Validation status	Regulatory/guideline status	Major limitations
Tier 1—clinically established	No representative Tier 1 liquid‐biopsy application identified	—	—	Gastric cancer	No liquid‐biopsy use case in the source evidence set met the Tier 1 definition used for this review.	—	No routine Tier 1 liquid‐biopsy use assigned in this table.	—
Tier 2—late‐stage clinical translation	Postoperative MRD and recurrence‐risk stratification	Tumor‐informed/longitudinal ctDNA MRD assays	Plasma	Resectable gastric/gastroesophageal junction adenocarcinoma after surgery or perioperative therapy	Prospective and longitudinal studies, together with meta‐analytic evidence, consistently associate postoperative ctDNA positivity with markedly higher recurrence risk [360, 361].	Prospective cohorts, Phase III substudy/longitudinal evidence and meta‐analysis	Investigational for MRD‐directed treatment decisions	Assay and timing heterogeneity; small‐to‐moderate cohorts; prognostic value is stronger than evidence that MRD‐guided therapy improves outcomes.
Tier 3—early‐stage clinical validation	Early diagnosis and nodal/peritoneal‐risk assessment	Exosomal miRNA panels and folate‐receptor‐positive CTCs	Plasma/serum EVs; peripheral blood	Early gastric cancer and perioperative risk assessment	Prospective studies support exosomal miRNA‐based nodal assessment and FR‐positive CTC associations with occult peritoneal metastasis/recurrence [338, 340].	Prospective single‐center clinical validation	Investigational	Small cohorts, limited external validation, nonspecific FR‐positive CTC biology, and assay‐specific cutoffs
Tier 3—early‐stage clinical validation	Molecular profiling and diagnostic/prognostic stratification	ctDNA multigene NGS, ctDNA methylation and EV‐DNA methylation	Plasma cfDNA; plasma EV‐DNA	Advanced/metastatic and all‐stage gastric cancer	VIKTORY and other clinical studies demonstrate molecular profiling feasibility; XAF1 and EV‐DNA methylation studies support prognostic/diagnostic validity [336, 355, 357, 384, 385].	Prospective umbrella‐trial experience plus retrospective/prospective cohorts	Investigational as a general liquid‐biopsy strategy; tissue remains central for many treatment‐defining biomarkers.	Not all detected alterations are actionable; tissue‐plasma discordance, small cohorts, and assay heterogeneity limit routine implementation.
Tier 3—early‐stage clinical validation	Exploratory diagnosis/prognosis/recurrence prediction	Exosomal miR‐17‐92, CTC‐WBC clusters, exosomal lncRNA H19 and gastric‐juice/exosomal methylation markers	Serum/plasma EVs; peripheral blood; gastric juice	Gastric cancer across stages	Single‐center clinical studies report associations with diagnosis, prognosis or recurrence [347, 363, 386, 387].	Retrospective or early prospective clinical validation	Investigational	Single‐center designs, small cohorts, nonstandardized sampling/normalization, and uncertain incremental value over endoscopy and pathology
Tier 4—discovery and mechanistic exploration	Mechanistic EV/ncRNA pathways	Linc‐ROR, circPTPN22, sEV‐circ6718 and related ncRNA pathways	EVs, tissue, cell lines and experimental models	Tumor progression, stromal remodeling and therapeutic‐target exploration	Mechanistic studies implicate EV/ncRNA pathways in gastric cancer growth and microenvironmental remodeling [380, 381, 388].	Mechanistic/preclinical with limited clinical correlation	Not clinically validated	Mechanistic findings do not establish liquid‐biopsy performance or clinical utility; external clinical validation is absent.

Abbreviations: cfDNA, cell‐free DNA; CTC, circulating tumor cell; ctDNA, circulating tumor DNA; EV, extracellular vesicle; GC, gastric cancer; GEJ, gastroesophageal junction; MRD, minimal residual disease; NGS, next‐generation sequencing. Entries are representative rather than exhaustive and are intended to mirror the principal use cases discussed in the corresponding manuscript section.

### 3.6 Nasopharyngeal Carcinoma

#### 3.6.1 Disease Background and Unmet Clinical Needs

Nasopharyngeal carcinoma (NPC) is a highly endemic epithelial malignancy closely associated with Epstein–Barr virus (EBV), with particularly high incidence in South China, Southeast Asia, and North Africa. In endemic regions, the incidence can reach 30.9 per 100,000 person‐years among males [389]. Although NPC is characterized by high radiosensitivity and favorable outcomes when treated at early stages, the majority of patients are diagnosed with locoregionally advanced disease, and locoregional recurrence and distant metastasis remain the primary causes of cancer‐related mortality [390, 391]. More than 20% of nonmetastatic NPC patients experience disease recurrence despite definitive chemoradiotherapy, highlighting persistent unmet clinical needs in early detection and posttreatment surveillance.

A major obstacle to improving NPC outcomes lies in its prolonged asymptomatic latent phase, during which malignant transformation occurs without overt clinical manifestations. Conventional screening and surveillance strategies are limited. Serological assays targeting EBV‐specific antibodies (e.g., IgA VCA, IgA EA, IgA EBNA‐1), although widely used, exhibit low PPVs, leading to high false‐positive rates and unnecessary follow‐up procedures [392, 393, 394]. Imaging‐based surveillance using MRI or CT is effective for detecting established recurrence but is resource‐intensive, costly, and poorly suited for long‐term population‐level monitoring. In addition, patient compliance with prolonged follow‐up protocols declines substantially over time, further compromising clinical effectiveness [393, 395].

These limitations have driven increasing interest in liquid biopsy approaches that can enable accurate, noninvasive, and scalable detection of NPC across the disease continuum. Given the viral etiology of NPC, EBV‐derived circulating biomarkers—together with tumor‐derived analytes—have emerged as uniquely powerful tools to address unmet needs in early screening, risk stratification, and dynamic disease monitoring.

#### 3.6.2 Early Detection and Screening

##### 3.6.2.1 cfEBV DNA‐Based Detection

Cell‐free EBV DNA (cfEBV DNA), released into the circulation from apoptotic or necrotic EBV‐infected NPC cells, represents the most clinically mature and extensively validated liquid biopsy analyte in NPC.

Plasma cfEBV DNA levels are significantly elevated in NPC patients compared with healthy individuals and those with benign nasopharyngeal diseases, supporting its diagnostic utility. Early landmark studies demonstrated cfEBV DNA detectability in over 95% of NPC patients but in fewer than 10% of healthy controls [396]. Subsequent investigations confirmed robust diagnostic performance, with reported sensitivities ranging from 68.8 to 100% and specificities of 88.0–100% [397, 398, 399, 400, 401]. Meta‐analyses further established plasma as the preferred specimen, outperforming serum in both sensitivity and specificity [402].

The diagnostic performance of cfEBV DNA is stage‐dependent, with increasing sensitivity as tumor burden rises. Large prospective screening studies in endemic regions have demonstrated that cfEBV DNA‐based screening identifies substantially higher proportions of early‐stage NPC compared with historical serology‐based approaches, suggesting the potential for earlier intervention; long‐term outcome data are still being accumulated [393, 397].

##### 3.6.2.2 cfEBV DNA Fragmentomics and Multiomics

Fragmentomics‐based analyses further refine risk stratification by identifying NPC‐specific cfEBV DNA features, including shortened fragment lengths, characteristic end‐motif patterns (CC‐depletion), and nucleosomal positioning, which together are associated with markedly increased NPC risk [403, 404]. A Cancer Cell 2025 study demonstrated that combined fragmentomics (FRAGMA: CGN/NCG) with cfDNA multiomics (NF + fragmentation + motifs + EBV content) achieved a relative risk of 87.1× (95% CI 35.2–215.8) for NPC. An integrated score achieved 99.9% AUC (98.15% sensitivity, 100% specificity in the case–control discovery cohort), with validation in larger prospective cohorts ongoing. EBioMedicine 2024 further validated these findings in prospective screening cohorts (*n* ∼ 20,000) [403, 405].

##### 3.6.2.3 Combined Methylation and ncRNA Strategies

To improve detection in early‐stage disease, combinatorial strategies have been developed. Integrating cfEBV DNA quantification with hypermethylation panels targeting tumor suppressor genes (e.g., RASSF1A, WIF1, DAPK1, RARβ2) significantly enhances sensitivity for Stage I–II NPC [399, 403].

DNA hypermethylation of tumor suppressor genes (e.g., CDKN2A, RASSF1A, CDH4, DAPK1) is an early event in NPC development, driven in part by EBV latent proteins that upregulate DNA methyltransferases [406, 407, 408, 409]. Hypermethylation markers have been detected in plasma and site‐specific samples, with higher sensitivity observed in nasopharyngeal swabs and rinsing fluids [410, 411]. RASSF1A methylation in nasopharyngeal swabs demonstrated 93% sensitivity and 100% specificity (AUC 0.956), with swab superior to plasma; dual‐gene SEPT9 + H4C6 achieved AUC 0.946.

EBV‐encoded miRNAs, predominantly from the *Bam*HI‐A rightward transcript (BART) family, are closely linked to NPC tumorigenesis. Circulating BART2‐5p miRNA demonstrated an AUC of 0.972 (Cohort 1) and 0.959 (Cohort 2), with preclinical sensitivity of 90.9% in high‐risk individuals [412, 413, 414, 415, 416, 417].

These epigenetic and ncRNA biomarkers complement cfEBV DNA by capturing early tumorigenic changes that may precede detectable viral load increases.

#### 3.6.3 Molecular Profiling and Therapy Selection

##### 3.6.3.1 cfEBV DNA for Staging and Risk Stratification

Beyond detection, cfEBV DNA serves as a robust biomarker for staging refinement. Integrating cfEBV DNA into conventional TNM staging systems improves risk stratification accuracy, enabling more precise identification of high‐risk patients for risk‐adapted management [418].

##### 3.6.3.2 Molecular Correlates

Molecular profiling of CTCs, including EBV‐associated features and tumor‐specific genetic alterations, supports potential utility in refining metastatic risk assessment. EGFR pathway alterations (PI3K/AKT/ROS) detected in EV cargo have been associated with metastatic potential [419].

#### 3.6.4 MRD Assessment and Recurrence Prediction

##### 3.6.4.1 cfEBV DNA Clearance Kinetics

cfEBV DNA dynamics are among the most extensively evaluated MRD‐related biomarkers in NPC. Postinduction chemotherapy (IC1/IC2) and post‐CCRT clearance kinetics, as well as “bounce” patterns (rebound after initial clearance), have been associated with recurrence risk and have been incorporated into risk‐adapted treatment studies.

In endemic cohorts (*n* = 1590; *n* = 673), post‐IC1 clearance was observed in 45.5% of patients, and a combined model achieved an AUC of 0.632 for risk stratification. Low‐risk patients receiving three cycles of IC achieved improved PFS (85.2 vs. 76.0%). In nonendemic cohorts (*n* = 167), post‐IC negative predictive value reached 92%. These findings support cfEBV DNA kinetics for recurrence‐risk stratification and for evaluating risk‐adapted induction‐chemotherapy strategies [420, 421].

##### 3.6.4.2 ctDNA Dynamic Monitoring

ctDNA dynamic monitoring has shown potential for recurrence prediction in research cohorts. The NPC‐DRIM model achieved a C‐index of 0.83. EP‐STAR (risk‐adapted treatment based on EBV DNA) demonstrated a 3‐year failure‐free survival of 89.1% (HR 0.41) in high‐risk patients receiving treatment intensification [422, 423, 424, 425]. These data provide emerging interventional evidence for EBV‐DNA‐informed risk‐adapted treatment in NPC, with applicability currently defined by the studied treatment setting.

##### 3.6.4.3 CTC‐Based Recurrence Prediction

CTCs are detectable across different stages of NPC progression and play a critical role in metastatic dissemination. In advanced and metastatic NPC, CTC enumeration correlates with disease burden and distant metastasis. Studies employing different isolation platforms—including size‐based systems (ISET, Microsieve) and marker‐based enrichment (CellSearch)—have reported variable detection rates and inconsistent correlations with plasma cfEBV DNA levels [426, 427, 428]. Despite these limitations, CTCs demonstrate high specificity for advanced disease and distant metastasis. Compared with plasma EBV DNA, CTCs exhibit superior specificity in distinguishing metastatic NPC, suggesting complementary roles for combined detection strategies [428].

#### 3.6.5 Treatment Response and Resistance Monitoring

##### 3.6.5.1 cfEBV DNA Dynamics During Therapy

Serial cfEBV DNA monitoring during and after treatment provides real‐time assessment of treatment response. Rising cfEBV DNA levels (“bounce” patterns) following initial clearance signal impending recurrence, often preceding radiographic detection by several months and providing an earlier molecular signal of disease relapse [418].

##### 3.6.5.2 Resistance Mechanisms

Emerging evidence has identified exosome‐mediated resistance mechanisms in NPC. Hypoxic exosomal PD‐L1 suppresses CD8^+^ T cell activity, facilitating immune evasion [429]. miR‐15a activates the γδT‐17/IL‐17/BCL‐2 pathway, promoting radioresistance [430]. circMYC has been implicated in radioresistance through ceRNA networks [431].

#### 3.6.6 Prognostic Stratification

##### 3.6.6.1 cfEBV DNA‐Based Prognosis

Elevated pretreatment cfEBV DNA levels correlate with inferior OS and PFS [432, 433]. Integrating cfEBV DNA into TNM staging improves risk stratification accuracy for identifying high‐risk patients [418].

##### 3.6.6.2 Methylation and circRNA‐Based Prognosis

Methylation panels including RASSF1A, WIF1, DAPK1, RARβ2, VILL (qMSP, 80.2% sensitivity, 100% specificity; combined with EBV DNA 94.8%), and FGF5 have demonstrated prognostic utility. A serum exosomal circRNA 8‐gene signature has been evaluated for prognostic prediction [422, 423, 424, 425].

##### 3.6.6.3 CTC and EV‐Based Prognosis

CTCs exhibit high specificity for advanced disease and distant metastasis [428]. Exosomal PTEN and cyclophilin A have been associated with RT/IT treatment response (AUC 1.00 for RT + IT response) [434]. EV‐miR‐197‐3p enhances radiosensitivity, suggesting prognostic potential [435].

#### 3.6.7 Emerging Biomarkers and Multianalyte Integration

##### 3.6.7.1 EV‐Mediated Mechanisms and Biomarkers

EGFR‐rich EVs activate PI3K/AKT signaling and suppress ROS, promoting metastasis [419]. FLT1‐enriched EVs promote angiogenesis [436]. LMP1–ALIX–PD‐L1 small EVs suppress CD8^+^ T cell activity, facilitating immune evasion [437]. Hypoxic exosomal PD‐L1 contributes to immunosuppression [429]. EV‐miR‐197‐3p enhances radiosensitivity [434, 435, 438, 439, 440].

##### 3.6.7.2 ncRNA Mechanisms

EBV‐encoded BART miRNAs (BART2‐5p, BART8‐3p, BART17‐5p, BART18‐5p) are closely linked to NPC tumorigenesis. BART8‐3p combined with EBV DNA achieved an AUC of 0.931 (88.9% sensitivity) [438]. IgA antibody triple panel (VCA/EA/EBNA1/Zta/Rta) achieved 96.2% sensitivity in NPC diagnosis [440].

##### 3.6.7.3 circRNA Networks

circMYC promotes radioresistance. A circRNA ceRNA network involving 314 differentially expressed genes and an 8‐gene model has been identified, with M1 macrophage upregulation and CD276/ICOSLG upregulation [425].

##### 3.6.7.4 Integrative Strategies

Integrated multianalyte strategies combining cfEBV DNA quantity, fragmentomic features, methylation signatures, and cellular biomarkers show superior diagnostic accuracy and predictive value compared with single‐analyte approaches.

#### 3.6.8 Evidence Maturity and Translational Status

Liquid biopsy applications in NPC span several levels of evidence maturity (Table 6), with no representative Tier 1—clinically established application identified under the framework used in this review. Tier 2—late‐stage clinical translation includes plasma cfEBV DNA kinetics and longitudinal monitoring for recurrence‐risk stratification and treatment‐response assessment, together with cfEBV DNA fragmentomics and integrated cfDNA multiomic approaches for early detection and future‐risk prediction. These applications are supported by multicohort, prospective, or independently validated clinical evidence, with risk‐adapted interventional evidence emerging for cfEBV DNA‐guided management. Tier 3—early‐stage clinical validation encompasses circulating EBV BART miRNAs, methylation‐based assays, serum exosomal circRNA signatures, and other circulating RNA‐ or EV‐based strategies evaluated in clinical cohorts for early detection, recurrence prediction, or prognostic stratification. Tier 4—discovery and mechanistic exploration comprises predominantly EV‐ and ncRNA‐associated mechanisms, including EGFR‐rich and FLT1‐enriched EVs, LMP1/PD‐L1‐related EV signaling, miR‐15a‐associated radioresistance, circMYC, and related circRNA networks. Overall, cfEBV DNA has the strongest translational evidence among NPC liquid‐biopsy analytes, particularly for screening and longitudinal risk assessment, whereas multianalyte, RNA‐, EV‐, and mechanistic approaches remain at earlier stages of clinical development.

**TABLE 6:** Evidence maturity of representative liquid biopsy applications in nasopharyngeal carcinoma.

Evidence tier	Clinical application	Representative analyte/assay strategy	Sample source	Disease context	Key representative evidence	Validation status	Regulatory/guideline status	Major limitations
Tier 1—clinically established	No representative Tier 1 liquid‐biopsy application identified	—	—	Nasopharyngeal carcinoma	cfEBV DNA is clinically important, but no application in this table is classified as a regulatory‐approved/guideline‐defined Tier 1 liquid‐biopsy companion diagnostic.	—	No Tier 1 application assigned in this framework.	—
Tier 2—late‐stage clinical translation	MRD/recurrence‐risk stratification and treatment‐response monitoring	Plasma cfEBV DNA kinetics/clearance and longitudinal ctDNA monitoring	Plasma	Locoregionally advanced NPC during induction chemoradiotherapy and follow‐up	Multicohort studies show that cfEBV DNA clearance and longitudinal dynamics provide prognostic and response information; prospective risk‐adaptive evidence is emerging [420, 421, 424].	Multicohort clinical validation with prospective interventional evidence emerging	Late‐stage translation; routine treatment escalation/de‐escalation based on cfEBV DNA dynamics remains indication‐specific and should not be inferred from prognostic validity alone.	Assay harmonization, threshold definition, endemic‐region generalizability, and treatment‐effect validation remain important.
Tier 2—late‐stage clinical translation	Early detection and future‐risk prediction	cfEBV DNA fragmentomics and integrated cfDNA multiomic signatures	Plasma cfDNA	NPC screening/high‐risk populations, especially endemic regions	Prospective cohort and independent‐validation studies show strong prediction using fragment size, end motifs and integrated cfDNA features [403, 405].	Prospective cohort plus independent clinical validation	Investigational	Evidence is concentrated in endemic populations; NGS cost, assay complexity and prospective downstream‐screening utility remain limitations.
Tier 3—early‐stage clinical validation	Early detection	Circulating EBV BART miRNAs	Plasma/serum	NPC screening and early detection, particularly in endemic/high‐risk populations	Circulating BART2‐5p and BART8‐3p have shown diagnostic signals in clinical cohorts [414, 438].	Retrospective/nested case–control and early clinical validation	Investigational	Limited prospective external validation; assay normalization and endemic‐region generalizability remain unresolved.
Tier 3—early‐stage clinical validation	Recurrence prediction and prognostic stratification	Plasma VILL/FGF5‐related methylation, serum exosomal circRNA, and other circulating EBV miRNAs	Plasma; serum EVs	Primary and postradiotherapy NPC	Clinical studies associate selected methylation and exosomal/circulating RNA signatures with recurrence or prognosis [423, 425, 438].	Prospective/retrospective clinical validation	Investigational	Single‐region and small‐cohort evidence; assay‐specific thresholds and independent validation are limited.
Tier 4—discovery and mechanistic exploration	Mechanistic metastasis, immune evasion and radioresistance	EGFR‐rich EVs, LMP1‐ALIX‐PD‐L1 small EVs, circMYC, and related pathways	EVs, tissue, cell lines and experimental models	NPC progression and radioresistance biology	Experimental studies define EV‐mediated metastatic and immunosuppressive pathways and ncRNA‐linked radioresistance [419, 430, 437].	Mechanistic/preclinical	Not clinically validated	No validated assay threshold, prospective clinical validation, or established decision pathway.

Abbreviations: cfDNA, cell‐free DNA; cfEBV DNA, cell‐free Epstein–Barr virus DNA; ctDNA, circulating tumor DNA; EV, extracellular vesicle; NPC, nasopharyngeal carcinoma; qPCR, quantitative polymerase chain reaction. Entries are representative rather than exhaustive and are intended to mirror the principal use cases discussed in the corresponding manuscript section.

## 4 Nononcologic Applications: Current Implementation and Emerging Translation

Liquid biopsy is most commonly discussed in oncology, but its conceptual and technical foundations extend well beyond cancer. Nononcologic applications are included here because they test the same core principles in biologically distinct settings: detection of low‐fraction signals in a high‐background biofluid, attribution of a circulating signal to its tissue or organism of origin, longitudinal measurement of dynamic biological processes, and translation of a molecular readout into a clinically meaningful action. In pregnancy, the minority signal is placental cfDNA in maternal plasma; after transplantation, it is donor‐derived cfDNA (dd‐cfDNA) released from the graft; in infection, it may be pathogen‐derived cfDNA; and in cardiovascular, neurological, autoimmune, and inflammatory disorders, the signal may arise from injured tissues or activated immune cells.

The maturity of these applications is highly uneven. NIPT is the clearest example of population‐scale implementation, with a defined biological target, large prospective validation studies, professional guidance, and established pathways for confirmatory testing. dd‐cfDNA in selected transplant settings and plasma microbial cfDNA sequencing have been evaluated as adjunctive tests in selected clinical settings, although their interpretation remains context dependent. By contrast, most cardiovascular, neurological, autoimmune, and inflammatory applications are supported primarily by proof‐of‐concept, case–control, or early prospective studies. This section therefore emphasizes clinical use cases, representative validation evidence, incremental value over existing workflows, and the remaining gap between analytical detectability and clinical utility rather than providing an exhaustive catalogue of candidate biomarkers.

### 4.1 Prenatal Testing: A Mature Population‐Scale Application of Low‐Fraction cfDNA Analysis

NIPT provides one of the clearest demonstrations that a low‐abundance circulating signal can become a population‐scale clinical test. Placental cfDNA, historically referred to as fetal cfDNA, originates predominantly from trophoblast turnover and is released into maternal plasma, where it is detected against a much larger background of maternal cfDNA. The discovery of fetal DNA in maternal plasma established the biological basis for noninvasive prenatal assessment and showed that liquid biopsy could achieve clinically consequential applications outside oncology [17].

The most mature indication is screening for common fetal aneuploidies, particularly trisomies 21, 18, and 13. Large prospective studies demonstrated that cfDNA‐based screening can achieve substantially higher discrimination for trisomy 21 than conventional first‐trimester screening while markedly reducing false‐positive results [441]. This clinical success is not attributable to analytical sensitivity alone. It reflects a favorable combination of a well‐defined target signal, sufficient prevalence for population screening, a clinically meaningful downstream decision, and an established confirmatory pathway using chorionic villus sampling or amniocentesis when a positive screening result may alter pregnancy management.

Professional guidance has therefore increasingly incorporated cfDNA‐based prenatal screening into routine counseling while maintaining a critical distinction between screening and diagnosis [442, 443]. A positive NIPT result should not be treated as definitive fetal diagnosis when an irreversible clinical decision is contemplated. PPV varies with the pretest probability of the condition, and discordant results may arise from confined placental mosaicism, a vanishing twin, maternal chromosomal abnormalities, maternal malignancy, low fetal fraction, or other biological sources of circulating DNA. These confounders are conceptually important for liquid biopsy as a whole because they demonstrate that high analytical accuracy does not eliminate uncertainty regarding signal origin.

The prenatal spectrum is also expanding beyond common aneuploidy screening. cfDNA can support noninvasive prenatal diagnosis of selected monogenic disorders by targeting paternally inherited or de novo variants, and more complex strategies can use relative haplotype dosage, targeted sequencing, or parental haplotyping [444]. Implementation studies in disorders such as cystic fibrosis illustrate that technically sophisticated prenatal cfDNA testing can be integrated into a diagnostic service, but uptake, assay design, inheritance pattern, availability of parental samples, and the need for disease‐specific validation create a very different evidence profile from common‐trisomy screening [445]. Similarly, expanded screening for sex‐chromosome aneuploidies or rare copy‐number disorders requires indication‐specific evaluation because lower prevalence can substantially reduce PPV even when analytical performance appears high.

NIPT therefore provides a benchmark for judging other liquid‐biopsy applications. Mature implementation requires more than detecting a low‐fraction signal: the test must have a defined intended use, validated performance in the relevant population, transparent counseling regarding residual risk and biological confounders, and an established pathway for confirmation and management. This combination of analytical validity, clinical validity, and actionable downstream workflow distinguishes clinically established NIPT from technically feasible but less mature expanded applications.

### 4.2 Organ Transplantation: Donor‐Derived cfDNA as a Marker of Allograft Injury

Organ transplantation represents a second setting in which liquid biopsy has progressed from molecular observation toward clinically integrated monitoring. Injured graft cells release donor‐derived nucleic acids into the recipient circulation, allowing the donor fraction or absolute amount of dd‐cfDNA to serve as a noninvasive readout of allograft injury. The underlying concept is attractive because tissue biopsy is invasive, repeated sampling is impractical, and conventional markers of organ function may change only after substantial injury has occurred.

The most mature evidence is in kidney transplantation. In the DART study, plasma dd‐cfDNA was evaluated against biopsy‐defined rejection and demonstrated that elevated donor DNA could identify active allograft injury, particularly antibody‐mediated rejection, while low values had potential value for ruling out active rejection in appropriate clinical contexts [446]. The key interpretive lesson, however, is that dd‐cfDNA is an injury marker rather than a rejection‐specific marker. Increased values can occur with ischemia–reperfusion injury, infection, recurrent disease, drug toxicity, surgical complications, and other forms of graft damage. Therefore, a positive result cannot by itself determine the cause of dysfunction or replace histopathology when tissue diagnosis is clinically required.

Heart transplantation provides a similar example of intermediate‐to‐high clinical maturity. A prospective multicenter study showed that dd‐cfDNA levels were associated with acute rejection and could be incorporated into surveillance alongside clinical assessment and endomyocardial biopsy [447]. In practice, the most defensible role is not stand‐alone diagnosis but risk stratification within a multimodal pathway that includes organ‐function measures, donor‐specific antibodies, imaging, and histopathology. This distinction is important because a molecular signal may be clinically useful even when it is not etiologically specific, provided that it changes the probability of clinically meaningful injury enough to alter the need or timing of further evaluation.

Evidence is also accumulating in lung transplantation, where chronic and acute allograft injury are difficult to characterize noninvasively. Longitudinal studies have linked higher dd‐cfDNA to allograft dysfunction, subsequent graft failure, and mortality, and clinical‐care cohorts have evaluated dd‐cfDNA as a composite marker of acute lung allograft dysfunction [448, 449]. Nevertheless, organ‐specific thresholds, the effects of infection and airway injury, and limited standardization across platforms currently restrict direct extrapolation from kidney or heart transplantation. Liver and other solid‐organ settings remain less standardized still, reinforcing the need for organ‐specific validation rather than a universal dd‐cfDNA cutoff.

Assay architecture also influences interpretation. Platforms may use donor‐recipient genotyping, predefined single‐nucleotide polymorphism panels, targeted sequencing, or digital PCR, and results can be reported as a donor fraction, absolute copy number, or related metrics. These methodological choices affect sensitivity, susceptibility to fluctuations in recipient cfDNA, turnaround time, and comparability across laboratories. Serial measurements may be more informative than isolated values because they provide an individual baseline and can distinguish persistent molecular injury from transient postprocedural elevation.

The next evidentiary step is therefore interventional rather than purely diagnostic. Prospective outcome data increasingly link recurrent or persistent dd‐cfDNA elevation with adverse graft outcomes, including kidney allograft loss, but association with outcome is not equivalent to proof that a dd‐cfDNA‐guided strategy improves that outcome [450]. Future trials should test predefined pathways in which a dd‐cfDNA result changes biopsy timing, immunosuppression, infection work‐up, or surveillance intensity and should determine whether those changes reduce unnecessary invasive procedures, prevent graft failure, improve survival, and remain cost effective. On current evidence, dd‐cfDNA is best regarded as a selectively implemented clinical adjunct whose value depends on integration with the surrounding transplant workflow.

### 4.3 Infectious Diseases: Microbial cfDNA and Host‐Response Liquid Biopsy

Infectious diseases illustrate a distinct liquid‐biopsy paradigm because the circulating target may be pathogen‐derived rather than human. Plasma microbial cfDNA sequencing can detect bacterial, viral, fungal, or parasitic DNA without requiring viable organisms or prior selection of a specific pathogen. Analytical and clinical validation studies have established that broad microbial cfDNA sequencing can identify diverse organisms from plasma, creating an alternative route to etiologic diagnosis when conventional culture is negative, slow, anatomically difficult to obtain, or affected by previous antimicrobial exposure [451].

The strongest current use cases are selected rather than universal. Multicenter clinical experience indicates that plasma microbial cfDNA can contribute diagnostically in difficult‐to‐diagnose infections, but its effect on management varies substantially according to pretest probability, specimen quality, the availability of conventional microbiology, and how clinicians interpret low‐level detections [452]. In febrile neutropenia and other immunocompromised populations, microbial cfDNA has been evaluated as a complement to blood culture, targeted PCR, and tissue sampling and may provide additional pathogen information when invasive sampling is difficult or unsafe [453]. Prospective pneumonia cohorts similarly suggest that plasma microbial cfDNA can add etiologic information in selected immunocompromised patients, although the incremental yield is not sufficiently uniform to justify indiscriminate testing [454, 455].

Interpretation remains one of the major barriers to routine use. Detection of microbial DNA does not necessarily prove that the detected organism is causing active disease. Signals can arise from transient DNAemia, contamination, colonization, commensal organisms, or clinically irrelevant low‐level microbial material. Conversely, false‐negative results can occur when pathogen burden is low, infection is compartmentalized, a pathogen is poorly represented in reference databases, or the causal organism releases little circulating DNA. For this reason, microbial cfDNA should complement rather than displace clinical assessment, imaging, site‐specific sampling, and conventional microbiology.

Resistance inference presents a second opportunity and limitation. Sequencing may identify genes associated with beta‐lactamase production, carbapenem resistance, vancomycin resistance, or other antimicrobial resistance mechanisms, but genotype does not always predict phenotypic susceptibility. The relevant resistance determinant may be carried by a different organism in a polymicrobial sample, expression may depend on regulatory context, and important mechanisms may be absent from the assay database. When viable organisms are available, culture‐based susceptibility testing therefore remains important even when resistance‐associated sequences are detected in plasma.

A complementary direction is analysis of the host response. Severe infection and sepsis increase host‐derived cfDNA through immune‐cell death, endothelial injury, tissue damage, and neutrophil extracellular trap formation. Plasma cfDNA concentration has been associated with disease severity and mortality in severe sepsis, supporting prognostic validity but not yet establishing that cfDNA‐guided treatment improves outcomes [456]. Tissue‐of‐origin analysis using methylation can extend this concept by identifying which cell types and organs contribute to the circulating DNA pool; in severe viral infection, methylation‐based deconvolution has demonstrated organ‐ and cell‐specific injury patterns associated with disease severity [457].

The most informative future infectious‐disease platform may therefore combine two questions: what organism is present, and what injury is occurring in the host? Such integration could theoretically distinguish etiologic diagnosis from systemic consequence, but the clinical endpoint must remain management relevant. Prospective studies should test whether microbial or host‐response cfDNA changes antimicrobial selection, reduces unnecessary invasive procedures, accelerates source control, improves organ support, or shortens hospitalization. Until those endpoints are demonstrated, microbial cfDNA is best positioned as a targeted adjunct for diagnostically challenging cases, whereas host‐response cfDNA remains predominantly early translational.

### 4.4 Cardiovascular Diseases: Tissue‐Injury Mapping Under Early Clinical Validation

Cardiovascular liquid biopsy seeks to measure molecular material released during cardiomyocyte death, endothelial injury, vascular inflammation, thrombosis, and fibrosis. Candidate analytes include total and mitochondrial cfDNA, cell‐type‐specific methylation patterns, fragmentomic features, EV cargo, circulating endothelial material, and cardiovascular RNAs. Among these approaches, tissue‐of‐origin inference using DNA methylation provides a particularly coherent biological framework because it can distinguish the cellular source of circulating DNA rather than relying only on total cfDNA concentration.

Proof‐of‐concept studies demonstrate the feasibility of this approach. Cardiomyocyte‐specific methylation patterns can identify cardiac‐cell death in circulating cfDNA, including elevations in settings of acute myocardial injury [458]. Broader human methylation atlases provide a reference framework for assigning cfDNA fragments to specific cell and tissue origins and have enabled analogous analyses across multiple organs [459]. Together, these studies establish biological and analytical validity for tissue‐injury mapping, but they do not by themselves establish incremental clinical utility over established cardiovascular diagnostics.

That distinction is particularly important in acute coronary syndromes. High‐sensitivity troponin is already sensitive, standardized, inexpensive, and embedded in validated diagnostic algorithms. Meta‐analytic evidence indicates that total cfDNA is elevated in acute myocardial infarction, but substantial between‐study heterogeneity, variable sampling times, and differences in assay methodology limit the immediate clinical interpretation of a single cfDNA value [460]. New liquid‐biopsy strategies must therefore answer a narrower question: do they identify myocardial injury earlier, distinguish etiologies that require different management, improve rule‐out or triage beyond existing biomarkers, or reveal tissue injury that conventional tests cannot resolve?

Vascular inflammation provides another biologically informative but less mature application. Neutrophil extracellular traps contribute extracellular DNA to the circulation and have been associated with thrombo‐inflammatory processes in ST‐elevation myocardial infarction; coronary NET burden and DNase activity have been related to infarct characteristics and reperfusion outcomes [461]. Such findings support mechanistic relevance but remain distinct from a validated diagnostic or treatment‐guiding assay. Similarly, EV and miRNA signatures can reflect endothelial dysfunction or myocardial stress, yet most remain at discovery or early clinical‐validation stages.

Accordingly, cardiovascular liquid biopsy should be judged against a high bar for incremental value. Prospective studies should prespecify sampling windows, comparator tests, decision thresholds, and management‐relevant endpoints, and should include the same patients in whom high‐sensitivity troponin, natriuretic peptides, echocardiography, cardiac magnetic resonance imaging, or coronary imaging would normally be used. At present, the field is best viewed as an emerging tissue‐injury mapping framework rather than a replacement for established cardiovascular biomarkers.

### 4.5 Neurological Diseases: Liquid Biopsy for Difficult‐to‐Access Tissues

Neurological disease is an attractive setting for liquid biopsy because direct sampling of brain tissue is rarely feasible and CSF collection is more invasive than peripheral blood sampling. The central challenge is therefore not merely detection, but recovery of sufficiently specific central‐nervous‐system‐derived signals from a circulation dominated by hematopoietic and peripheral‐tissue material. Candidate readouts include brain‐derived cfDNA, neuronal or glial EV cargo, brain‐enriched RNAs, and tissue‐specific methylation signatures [462].

Neurodegenerative disorders illustrate both the opportunity and the current limitations. In late‐onset Alzheimer's disease, circulating cfDNA 5‐hydroxymethylcytosine signatures have been evaluated as diagnostic biomarkers, demonstrating that disease‐associated epigenetic information can be detected in peripheral blood [463]. Neuronal‐derived EV biomarkers have also been associated with longitudinal cognitive decline, supporting the concept that cell‐type‐enriched vesicular cargo can reflect ongoing neurodegenerative processes [464]. These studies establish disease association and biological plausibility, but routine diagnostic utility requires comparison with rapidly maturing plasma and cerebrospinal‐fluid protein biomarkers, neuroimaging, and established clinical criteria.

Tissue‐specific methylation offers a complementary strategy because it can identify the cellular origin of cfDNA independently of a disease‐specific mutation. Proof‐of‐concept work has detected oligodendrocyte‐derived DNA in relapsing multiple sclerosis and neuronal or glial cfDNA after acute neurological injury, demonstrating that tissue‐specific cell death can be reconstructed from circulation [465]. This framework is especially relevant when the clinical question concerns compartment‐specific tissue damage rather than systemic inflammation alone.

EV approaches may further improve cell‐type specificity. For example, blood EV populations enriched for microglial markers have been investigated in multiple sclerosis, illustrating the possibility of isolating CNS‐relevant vesicular signals from peripheral blood [466]. However, EV isolation, marker specificity, platform heterogeneity, and the absence of standardized reference materials remain substantial barriers. A biomarker that correlates with disease activity in a selected cohort is not yet equivalent to a clinically validated assay for diagnosis, relapse prediction, or treatment escalation.

Acute neurological injury, including stroke and traumatic brain injury, provides another potential use case because molecular release can occur rapidly after tissue damage. Here, clinically meaningful studies should ask whether a liquid‐biopsy signal improves triage, identifies patients requiring urgent imaging or intervention, distinguishes tissue injury patterns, or predicts complications beyond current neurological examination and imaging. Standardized sampling times are particularly important because the kinetics of cfDNA and EV release may be rapid and highly time dependent.

Overall, most nononcologic neurological applications remain in discovery or early clinical validation. Their strongest conceptual advantage is access to otherwise difficult‐to‐sample tissue, but the field must demonstrate specificity of tissue origin, reproducibility across platforms, and incremental value over existing protein biomarkers and imaging. Prospective longitudinal studies, especially in presymptomatic or early disease, will be required before blood‐based CNS liquid biopsy can be considered clinically established.

### 4.6 Autoimmune and Inflammatory Diseases: Immune‐Cell Turnover and Target‐Organ Injury

Autoimmune and inflammatory diseases provide a setting in which liquid biopsy may capture two biologically distinct processes: systemic immune activation and injury to the organs targeted by that immune response. Candidate signals include immune‐cell‐derived cfDNA, neutrophil extracellular trap‐associated DNA, EV cargo, circulating miRNAs, and tissue‐specific methylation patterns. This distinction is clinically relevant because conventional inflammatory markers often indicate the presence of inflammation without specifying which immune compartments are active or which organs are undergoing cell death.

Systemic lupus erythematosus (SLE) provides a representative example. Higher circulating cfDNA levels have been associated with active disease, supporting cfDNA as a marker of immune‐cell turnover and inflammatory activity [467]. Combined studies of plasma cfDNA and circulating miRNAs have likewise explored their relationship with SLE activity [468]. These associations are biologically plausible because apoptosis, impaired clearance of nucleic acids, and NET formation are central features of lupus biology. However, most studies remain observational, and it is not yet established that cfDNA measurements improve treatment decisions beyond established clinical indices, complement levels, anti‐dsDNA antibodies, organ‐specific laboratory tests, and imaging or biopsy when required.

A more informative direction may be deconvolution of the cellular source of cfDNA. Immune‐derived methylation signatures can reveal turnover of specific immune‐cell populations and thereby expose remote immune processes that are not apparent from total cfDNA concentration [469]. In parallel, tissue‐of‐origin methylation frameworks can identify cell death from organs such as the liver, pancreas, nervous system, or other tissues [459, 465]. For multisystem autoimmune disease, combining these dimensions could in principle separate systemic immune activation from target‐organ injury, which is more clinically relevant than a nonspecific rise in total circulating DNA.

The main translational barriers are disease heterogeneity, fluctuating activity, treatment exposure, infection, comorbidity, and age‐related hematopoietic changes. These factors can alter cfDNA concentration and composition independently of autoimmune activity and can generate apparently strong case–control discrimination that does not persist in longitudinal practice. Similarly, EV and miRNA signatures often depend on isolation methods, normalization strategies, and small discovery cohorts, limiting cross‐study reproducibility.

Future studies should therefore focus on defined clinical decisions: whether a liquid‐biopsy profile can identify impending organ flare, distinguish inflammatory activity from irreversible damage, reduce the need for invasive biopsy, or support safer escalation or de‐escalation of immunosuppression. Repeated within‐patient sampling may be particularly valuable because each patient can serve as a biological baseline. Until such use cases are prospectively validated, immune‐cell and tissue‐of‐origin liquid biopsy in autoimmune disease should be regarded as an early translational framework rather than a routine clinical test.

### 4.7 Evidence Maturity and Clinical Readiness Across Nononcologic Applications

Across nononcologic settings, the trajectory from analytical performance to routine care is highly heterogeneous. NIPT for common aneuploidy screening has reached the highest maturity because it combines a defined low‐fraction biological signal with large‐scale validation, professional guidance, counseling standards, confirmatory diagnostic pathways, and clear consequences for clinical management. dd‐cfDNA in kidney and heart transplantation and plasma microbial cfDNA sequencing in selected difficult infections occupy an intermediate position: both can provide clinically useful adjunctive information, but neither should be treated as a universal stand‐alone replacement for biopsy, conventional microbiology, or other established diagnostic tools.

Cardiovascular, neurological, autoimmune, and inflammatory applications remain substantially earlier in translation. In these fields, tissue‐of‐origin methylation, cfDNA kinetics, EV cargo, and immune‐cell‐derived signals have established important biological concepts and, in some cases, early clinical validity. The next step is not simply to improve AUC or analytical sensitivity, but to demonstrate incremental value over strong existing standards, specify when the test should be ordered, define what action follows a positive or negative result, and show that the resulting pathway improves outcomes or reduces harm.

A cross‐system comparison suggests three distinct translational archetypes. First, NIPT illustrates a screening model in which the biological source, target condition, testing population, and confirmatory pathway are sufficiently well defined to support broad implementation. Second, dd‐cfDNA and microbial cfDNA illustrate an adjunctive‐monitoring model: the molecular signal can refine risk or accelerate diagnosis, but interpretation remains conditional on conventional clinical, imaging, microbiological, immunological, or histopathological information. Third, cardiovascular, neurological, autoimmune, and inflammatory applications illustrate a discovery‐to‐validation model in which tissue‐of‐origin signals and molecular kinetics are biologically informative but have not yet demonstrated consistent incremental value over established standards. These archetypes emphasize that clinical maturity depends as much on the decision pathway surrounding a test as on the analyte or platform itself.

Table 7 summarizes representative nononcologic use cases using the same evidence‐maturity logic applied to oncology. The tiers are assigned to a specific clinical use case and assay strategy rather than to an analyte itself because the same analyte can have different levels of maturity in different settings. This cross‐system comparison also reinforces the broader theme of the review: a circulating molecular signal becomes clinically meaningful only when analytical validity, clinical validity, clinical utility, workflow feasibility, and downstream decision‐making are aligned. These principles provide the bridge from the current state of nononcologic liquid biopsy to the translational priorities discussed in Section 5.

**TABLE 7:** Clinical readiness and evidence maturity of representative nononcologic liquid biopsy applications.

Evidence tier	Clinical application	Representative analyte/assay strategy	Sample source	Disease context	Key representative evidence	Validation status	Regulatory/guideline status	Major limitations
Tier 1—clinically established	Common fetal aneuploidy screening	Placental cfDNA; counting/SNP‐based NIPT	Maternal plasma	Pregnancy; screening for common trisomies	The discovery of placental cfDNA established the biological basis, and large prospective validation plus professional guidance support population‐scale screening [17, 441, 442, 443].	Population‐scale clinical implementation	Clinically established as screening; positive results require confirmatory diagnostic testing.	Screening, not diagnosis; placental mosaicism, vanishing twin, low fetal fraction, and maternal findings can cause discordance.
Tier 2—late‐stage clinical translation	Selected monogenic noninvasive prenatal diagnosis	Targeted variant/haplotype analysis of placental cfDNA	Maternal plasma	High‐risk pregnancies for selected inherited disorders	Clinical service experience demonstrates feasibility for selected monogenic disorders [444, 445].	Indication‐specific implementation in specialized centers	Late‐stage translation/specialized clinical implementation rather than population‐wide use.	Disease‐specific assay design, inheritance pattern, parental haplotypes, and limited scalability
Tier 2—late‐stage clinical translation	Kidney allograft injury/rejection‐risk assessment	Donor‐derived cfDNA fraction or absolute amount	Plasma	Kidney transplantation	Biopsy‐referenced and outcome‐based studies support dd‐cfDNA as an adjunctive marker of allograft injury [446, 450].	Prospective/multicenter validation; selective clinical implementation	Adjunctive clinical use in selected settings; not a stand‐alone etiologic diagnosis of rejection.	Not rejection‐specific; platform/threshold variability; management utility and biopsy‐reduction strategies remain context dependent.
Tier 2—late‐stage clinical translation	Heart allograft surveillance	Donor‐derived cfDNA	Plasma	Heart transplantation	A prospective multicenter study linked dd‐cfDNA to graft injury/rejection and supported noninvasive surveillance [447].	Prospective multicenter validation; selective implementation	Adjunctive surveillance in selected settings	Requires integration with organ function, immunologic data, and biopsy; not etiologically specific
Tier 3—early‐stage clinical validation	Lung allograft dysfunction	Donor‐derived cfDNA	Plasma	Lung transplantation	Longitudinal and clinical‐care cohorts associate dd‐cfDNA with allograft dysfunction, graft failure and mortality [448, 449].	Early clinical validation/selective use	Not standardized for routine universal use	Infection and airway injury confound interpretation; organ‐specific thresholds and platform harmonization are lacking
Tier 2—late‐stage clinical translation	Difficult‐to‐diagnose infection in selected patients	Plasma microbial cfDNA metagenomic sequencing	Plasma	Culture‐negative, deep‐seated, or immunocompromised infection	Analytical validation, multicenter studies, and prospective cohorts show added etiologic information in selected diagnostic pathways [451, 452, 453, 454, 455].	Clinical validation with selective implementation	Selective adjunctive use; clinical value is highest when conventional microbiology is limited.	Contamination/colonization, database coverage, variable incremental yield, cost, and uncertain effect on outcomes.
Tier 3—early‐stage clinical validation	Sepsis severity and host‐injury profiling	Total host cfDNA and tissue‐of‐origin methylation	Plasma	Sepsis and severe systemic infection	Observational studies link cfDNA to prognosis, while methylation‐based tissue‐of‐origin approaches resolve organ injury [456, 457].	Observational/early prospective clinical validation	Investigational	Association does not establish treatment utility; inflammatory states are nonspecific and kinetics are time sensitive.
Tier 3—early‐stage clinical validation	Cardiomyocyte injury/tissue‐of‐origin mapping	Cardiomyocyte‐specific cfDNA methylation and total cfDNA	Plasma	Acute myocardial injury and cardiovascular disease	Proof‐of‐concept human studies and meta‐analytic evidence support detection of cardiomyocyte death [458, 459, 460].	Early clinical validation	Investigational	Must demonstrate incremental value beyond high‐sensitivity troponin, imaging, and established risk models; timing and assays vary
Tier 4—discovery and mechanistic exploration	Thrombo‐inflammatory vascular injury	NET‐associated extracellular DNA	Plasma/coronary samples	Acute coronary syndromes and vascular inflammation	Mechanistic and observational studies link NET burden to infarct biology and outcomes [461].	Discovery/mechanistic clinical research	Not clinically validated	No standardized threshold, limited specificity, and no demonstrated test‐guided intervention
Tier 3—early‐stage clinical validation	Neurodegeneration/CNS‐derived signal detection	cfDNA epigenetic signatures and neuronal/glial EVs	Plasma/serum	Alzheimer disease and related neurodegenerative disorders	Disease‐association and longitudinal studies show that peripheral biofluids can carry CNS‐relevant molecular signals [462, 463, 464].	Early clinical validation	Investigational.	Very low abundance, uncertain cell of origin, and need to demonstrate value beyond validated plasma/CSF protein biomarkers and imaging
Tier 3—early‐stage clinical validation	Acute neurological injury and demyelinating disease	Tissue‐specific cfDNA methylation and microglial EVs	Plasma/serum	Acute CNS injury and multiple sclerosis	Tissue‐of‐origin and disease‐specific studies support neuronal/glial injury mapping and early biomarker development [465, 466].	Early clinical validation	Investigational	Time‐sensitive kinetics, disease heterogeneity, assay standardization, and prospective decision thresholds are unresolved.
Tier 3—early‐stage clinical validation	Autoimmune disease activity/immune‐cell turnover	Total cfDNA, immune‐derived cfDNA and circulating miRNAs	Plasma/serum	Systemic lupus erythematosus and related inflammatory disorders	Clinical studies and immune‐cell methylation frameworks support association with disease activity and immune‐cell turnover [467, 468, 469].	Observational/early clinical validation	Investigational	Disease and treatment heterogeneity, infection confounding, and limited evidence that testing changes immunosuppressive management
Tier 4—discovery and mechanistic exploration	Target‐organ injury mapping in multisystem inflammation	Tissue‐of‐origin cfDNA methylation	Plasma	Multisystem inflammatory disease	Cell‐type methylation atlases and proof‐of‐concept cell‐death studies provide a framework for separating systemic immune activation from organ‐specific injury [459, 465, 469].	Discovery/mechanistic exploration	Not clinically validated	Reference atlases, disease‐specific thresholds, longitudinal validation, and utility trials are required.

Abbreviations: cfDNA, cell‐free DNA; CNS, central nervous system; dd‐cfDNA, donor‐derived cell‐free DNA; EV, extracellular vesicle; NET, neutrophil extracellular trap. Entries are representative rather than exhaustive and are intended to mirror the principal use cases discussed in the corresponding manuscript section; NIPT, noninvasive prenatal testing; SLE, systemic lupus erythematosus.

## 5 Future Directions: From Biomarker Discovery to Clinically Actionable Liquid Biopsy

The next phase of liquid biopsy will be defined less by the accumulation of additional biomarkers than by disciplined translation of molecular signals into clinically actionable workflows. Across oncology and nononcologic medicine, the central challenge is moving from analytical detectability to evidence that a test improves decisions or outcomes. Progress therefore depends on use‐case‐specific assay design, explicit evidence grading, prospective validation, integration with established diagnostic pathways, transparent computational interpretation, and implementation strategies that are reproducible and scalable.

### 5.1 Use‐Case‐Driven Development and Evidence Maturity

No liquid‐biopsy analyte is universally optimal. cfDNA, ctDNA, CTCs, EVs, cfRNA, ncRNAs, TEPs, microbial cfDNA, and tissue‐specific methylation signals capture different biological dimensions and have distinct vulnerabilities. Assay development should therefore begin with the clinical question. Early‐detection tests require very high specificity, reliable tissue‐of‐origin inference, acceptable false‐positive rates, and safe downstream diagnostic pathways; therapy‐selection assays require actionable alterations to be detected with sufficient sensitivity and turnaround time; MRD assays must connect molecular positivity with a defined treatment or surveillance decision; and nononcologic assays must show that earlier molecular detection adds value beyond established diagnostic tools.

Evidence maturity should be reported separately from analytical performance. Analytical validity asks whether an assay measures its intended signal accurately and reproducibly; clinical validity asks whether that signal is associated with disease presence, burden, prognosis, or response; and clinical utility asks whether using the result improves patient management or outcomes. Sensitivity, specificity, AUC, detection rate, and lead time are important performance measures, but they do not by themselves establish clinical utility.

Future studies should therefore move beyond case–control discrimination and incorporate predefined clinical decision points and meaningful endpoints. Depending on the use case, these may include stage shift, recurrence‐free or OS, time to appropriate therapy, avoidance of invasive procedures, treatment toxicity, patient‐reported outcomes, cost per clinically meaningful diagnosis, and health‐system feasibility. Explicitly linking assay performance to the intended action will also make evidence grading more interpretable across diseases and platforms.

### 5.2 MRD and MCED: From Molecular Detection to Clinical Utility

MRD and MCED represent two of the most consequential translational frontiers for liquid biopsy. Both operate in low‐signal settings and therefore benefit from increasingly sensitive molecular approaches, but both also illustrate why detection alone is insufficient: the decisive questions concern what clinical action follows a positive or negative result and whether that action ultimately benefits patients.

#### 5.2.1 MRD and Recurrence Monitoring: Tumor‐Informed and Tumor‐Naive Strategies

Postoperative or posttreatment ctDNA positivity frequently identifies patients at high risk of recurrence and may precede radiographic relapse by months [239, 242, 470]. The major translational question, however, is whether MRD‐guided escalation, de‐escalation, or surveillance improves outcomes. Future MRD trials should therefore be designed around a defined intervention rather than treating molecular detection as an endpoint in itself.

Two broad strategies are being developed. Tumor‐informed assays first identify patient‐specific tumor variants and then track them in plasma, which can improve specificity for known tumor‐derived alterations but requires adequate tissue, individualized assay design, additional turnaround time, and logistical infrastructure. Tumor‐naive assays do not require prior tumor sequencing and may be more scalable, but they face greater difficulty distinguishing low‐level tumor signals from background cfDNA, clonal hematopoiesis, sequencing noise, and benign tissue injury.

The relative value of these strategies is likely to depend on tumor type, stage, treatment setting, sampling time, assay limit of detection, marker selection, integration of methylation or fragmentomic features, and the intended clinical action. A test used to support treatment de‐escalation, for example, requires particularly strong confidence in a negative result, whereas a test used to trigger intensified surveillance or clinical‐trial enrollment may tolerate different performance trade‐offs.

Clonal hematopoiesis is a major confounder for low‐variant‐allele‐fraction mutation detection, especially in older individuals and in patients exposed to chemotherapy or chronic inflammatory states [471, 472]. Paired white‐blood‐cell sequencing, tissue‐of‐origin inference, fragmentomic features, longitudinal kinetics, conservative reporting thresholds, and orthogonal validation can help mitigate this problem and should be incorporated as appropriate into MRD assay development and reporting.

The design of MRD utility trials should reflect the clinical asymmetry of escalation and de‐escalation decisions. Escalation trials must determine whether intervening on molecular positivity improves recurrence‐free or OS sufficiently to justify additional treatment toxicity, whereas de‐escalation trials require strong assurance that a negative result can safely support less treatment or less intensive surveillance. Assay timing, repeat‐testing rules, indeterminate results, and management of discordance with imaging or pathology should therefore be prespecified. Biomarker‐stratified observational cohorts can establish prognostic validity, but interventional evidence is ultimately required when the intended claim is that MRD testing should change therapy.

#### 5.2.2 MCED: Integrating Methylation, Fragmentomics, and Multianalyte Signals

MCED aims to detect cancer signals across multiple tumor types from a single blood sample and, ideally, predict the tissue or organ of origin. Methylation profiling has become a leading strategy because DNA methylation patterns are tissue specific and are frequently altered during tumorigenesis [21, 473]. Fragmentomics, nucleosome positioning, end motifs, copy‐number features, proteins, metabolites, and EV‐associated signals may provide complementary information [474, 475].

Implementation requires particular caution because MCED is intended for largely asymptomatic populations with low disease prevalence. Even a small false‐positive rate can generate substantial downstream imaging, endoscopy, anxiety, cost, and potential harm, while false‐negative results may create inappropriate reassurance. Overdiagnosis, detection of indolent disease, uncertainty in cancer‐signal origin, and variable sensitivity across tumor types and stages must therefore be evaluated alongside headline performance metrics.

The central evidence gap is clinical utility. MCED should be evaluated not only by cancer‐signal detection but by whether testing produces a meaningful stage shift, reduces cancer‐specific mortality, avoids unnecessary diagnostic procedures, and remains cost effective in real health systems. Positive results also require standardized follow‐up pathways integrating tissue‐of‐origin prediction, targeted imaging or endoscopy, clinical risk factors, and existing screening programs. MCED is therefore best positioned as a potential complement to established screening rather than an immediate replacement.

Future platforms will likely integrate multiple signal classes and clinical risk factors. Such integration may improve sensitivity or tissue‐of‐origin inference, but it also increases model complexity, cost, and susceptibility to overfitting or population bias. Large, diverse, prospective validation cohorts and clearly specified downstream actions will be essential before multianalyte MCED can be judged by clinical rather than analytical success.

MCED evaluation likewise requires trial designs that capture the full diagnostic cascade rather than the blood test alone. Relevant endpoints include the proportion of cancers diagnosed at potentially curable stages, time from positive test to definitive diagnosis, number and invasiveness of follow‐up procedures, interval cancers after negative testing, overdiagnosis, and cancer‐specific outcomes. Because these endpoints depend on background screening programs and healthcare infrastructure, evidence generated in one system may not transfer directly to another. The clinical value of MCED will therefore depend on both assay performance and a reproducible pathway for resolving the signal of origin and completing diagnostic work‐up without unacceptable downstream burden.

### 5.3 AI‐Assisted, Longitudinal, and Multimodal Interpretation

As liquid‐biopsy assays become more multidimensional, computational interpretation increasingly becomes part of the test rather than a downstream convenience. AI and machine‐learning models can integrate mutations, methylation, fragmentomic features, EV cargo, protein markers, imaging, pathology, and clinical variables for tissue‐of‐origin classification, MRD risk modeling, response prediction, resistance detection, and multimodal decision support. These opportunities also introduce risks related to transportability, calibration, interpretability, and bias.

Models trained in narrow populations may perform poorly when disease prevalence, ancestry, platform, preanalytical handling, or clinical setting changes. Future AI‐enabled assays should therefore undergo external validation, population‐level calibration, transparent reporting, and prospective evaluation of clinical utility. When model outputs trigger invasive procedures, treatment escalation, or changes in surveillance, reports should quantify uncertainty and clarify the features contributing to the prediction. For clinical translation, model evaluation should consider robustness, bias, interpretability, cybersecurity, data provenance, and postdeployment performance in addition to accuracy.

For AI‐enabled liquid biopsy, prospective validation should also distinguish model discrimination from decision performance. A model may retain a high area under the receiver‐operating‐characteristic curve yet become poorly calibrated when deployed in a population with different prevalence, ancestry, comorbidity, or sample‐processing characteristics. Validation should therefore assess calibration, clinically relevant thresholds, subgroup performance, failure modes, and the consequences of false‐positive and false‐negative outputs. Where models are updated after deployment, version control and predefined monitoring for performance drift are essential so that changes in the computational component do not silently alter the clinical meaning of the assay.

Longitudinal interpretation is a second priority. Many circulating analytes have short half‐lives and are inherently suited to serial monitoring, yet single time points and fixed thresholds remain common. Future algorithms should incorporate individual baselines, biological variation, molecular kinetics, treatment timing, and clinical context. In oncology, longitudinal ctDNA may distinguish molecular response, persistent residual disease, emerging resistance, and impending relapse; in transplantation, trends in dd‐cfDNA may be more informative than isolated values; and in infection, microbial cfDNA kinetics may contribute to response assessment.

More frequent measurement is not inherently beneficial. Abnormal molecular trends must be connected to evidence‐based confirmatory testing and management pathways, with prespecified sampling intervals and action thresholds. Otherwise, serial testing may increase false alarms, cost, and patient anxiety without improving outcomes.

Liquid biopsy should also be interpreted within multimodal clinical workflows. Tissue pathology remains essential for histologic diagnosis and many biomarker assessments, imaging provides anatomical localization and staging, and clinical variables determine pretest probability and management options. Liquid biopsy adds dynamic molecular information, but its value depends on how that information changes decisions within this broader framework. Future workflows should therefore specify when to test, which sample and assay to use, how positive and negative results are interpreted, when testing is repeated, and what action follows.

The most useful multimodal strategies are therefore likely to be selective rather than maximally complex. Adding imaging, pathology, or clinical risk variables should be justified by incremental information beyond the liquid‐biopsy signal alone and by a clear effect on the subsequent decision. This principle is particularly important for low‐prevalence screening and MRD settings, where small gains in discrimination may be outweighed by added cost, workflow complexity, or false‐positive downstream testing. Prospective decision‐support studies should compare integrated workflows with current standards of care rather than evaluating each molecular or computational component in isolation.

### 5.4 Emerging Platforms, Multiomics, and Standardization

Technological development remains essential, particularly for signals that are rare, heterogeneous, or poorly captured by bulk assays. Single‐cell analysis of CTCs, single‐EV profiling, high‐resolution cfDNA methylation, long‐read sequencing, microfluidic enrichment, and lab‐on‐a‐chip platforms may improve sensitivity, reduce sample requirements, preserve biological context, or support point‐of‐care workflows [476, 477, 478]. Most of these approaches, however, remain at the research or early clinical‐validation stage.

Translation requires reproducible manufacturing, standardized sample processing, robust quality control, interlaboratory comparability, and evidence of clinical utility. EV‐based assays must address vesicle heterogeneity, isolation bias, contamination, inconsistent nomenclature, and lack of universal reference materials, whereas CTC platforms must contend with rarity, epithelial–mesenchymal plasticity, and variable capture efficiency. These technical constraints should be treated as central determinants of evidence maturity rather than secondary engineering details.

Multiomic integration offers the possibility of combining genomic, epigenomic, fragmentomic, transcriptomic, proteomic, metabolomic, and imaging‐derived information. Such integration may improve biological resolution, but greater dimensionality also increases cost, model complexity, and risk of overfitting. Multiomic platforms should therefore be evaluated against clinically meaningful endpoints and independent validation cohorts rather than discovery‐set performance alone.

The future standardization agenda should build on the preanalytical principles outlined in Section 2.6 rather than duplicate them. Priority areas include cross‐platform reference materials, external quality assessment, harmonized reporting, and transparent specification of assay performance. Reports should state the limit of detection and limit of blank, relevant molecular fractions, quality‐control metrics, positivity criteria, and, where appropriate, clonal‐hematopoiesis filtering or tissue‐of‐origin uncertainty. AI‐assisted tests should additionally report model version, validation population, intended use, and performance‐monitoring strategy. These requirements will be increasingly important as liquid biopsy moves beyond ctDNA toward EVs, cfRNA, fragmentomics, methylation, and integrated multiomics.

### 5.5 Real‐World Implementation and Responsible Translation

Clinical implementation ultimately requires evidence of value, not only technical feasibility. Cost effectiveness depends on disease prevalence, test performance, downstream diagnostic workup, availability of effective interventions, avoided procedures, treatment effects, and health‐system context. The relevant value proposition is therefore use‐case specific: NIPT can reduce invasive prenatal testing in appropriate populations; dd‐cfDNA may support earlier graft‐injury evaluation and reduce biopsy in selected settings; microbial cfDNA may shorten time to an etiologic diagnosis, whereas the value of MCED and MRD will depend heavily on whether test‐guided management improves outcomes.

Health‐economic evaluation should be embedded prospectively into late‐stage validation rather than added only after technical development is complete. Relevant analyses should capture not only the price of the assay but also repeat sampling, confirmatory imaging or biopsy, specialist visits, treatment changes, avoided procedures, downstream complications, and the opportunity cost of additional diagnostic work. The appropriate economic endpoint will differ by use case: cost per clinically meaningful diagnosis may be relevant for screening, whereas transplant surveillance may be judged by biopsies avoided or graft outcomes preserved, and MRD by the balance between recurrence prevented and unnecessary systemic therapy delivered. Reimbursement frameworks should therefore be tied to a defined intended use and demonstrated clinical value rather than to the technological sophistication of the platform itself.

Equitable access should be considered during development rather than after deployment. Assays that require high‐cost sequencing, specialized infrastructure, or narrowly trained models may widen health disparities if they are available only in resource‐intensive centers or perform poorly in underrepresented populations. Diverse validation cohorts, scalable laboratory workflows, transparent reimbursement policies, and attention to health‐system capacity are therefore integral to translation.

Real‐world implementation also requires continued performance surveillance after adoption. Laboratory drift, changes in patient mix, new therapies, evolving screening programs, and software updates can alter predictive value even when the underlying assay is analytically stable. Registries and postimplementation audits should therefore track test utilization, discordant results, downstream procedures, time to treatment, patient outcomes, and distribution of access across demographic and geographic groups. Such evidence can identify when a test is being used outside its validated indication and can inform recalibration, guideline refinement, or withdrawal of low‐value use.

Taken together, future progress should be judged by whether liquid biopsy becomes more clinically reliable, interpretable, and actionable rather than simply more sensitive or more complex. The priorities emerging from these sections—use‐case definition, explicit evidence grading, prospective demonstration of clinical utility, control of biological confounders, multimodal integration, standardization, responsible AI, and equitable implementation—provide the basis for the concrete translational priorities summarized in the Conclusion.

## 6 Conclusion and Translational Priorities

Over the past two decades, liquid biopsy has evolved from the detection of circulating nucleic acids into a broad molecular framework spanning ctDNA, CTCs, EVs, circulating RNAs, TEPs, microbial nucleic acids, donor‐derived cfDNA, and tissue‐of‐origin signals. This expansion has been accompanied by a shift from mutation‐centric testing toward methylation, fragmentomics, transcriptomic and proteomic readouts, longitudinal kinetics, and multianalyte integration. The resulting field is no longer defined by a single specimen type or platform, but by the ability to recover biologically informative signals from accessible biofluids and connect them to a specific clinical question.

The present evidence, however, is markedly heterogeneous. Plasma genotyping for selected actionable alterations and NIPT demonstrate that liquid biopsy can achieve clinically established use when analytical validity, clinical validity, intended population, downstream action, and implementation pathway are aligned. MRD, MCED, dd‐cfDNA, microbial cfDNA, and many emerging EV‐, RNA‐, TEP‐, methylation‐, and fragmentomic approaches occupy different intermediate stages of translation. Across these applications, high sensitivity, specificity, AUC, or lead time should not be interpreted as clinical utility by themselves. The decisive standard is whether testing improves a defined decision, patient outcome, safety, efficiency, or value relative to existing care.

Several priorities should therefore define the next stage of the field. First, assay development should begin with a clearly specified clinical use case and evidence tier rather than with biomarker accumulation. Second, MRD and other longitudinal applications require prospective interventional trials that test predefined escalation, de‐escalation, or surveillance strategies. Third, MCED and multianalyte early‐detection platforms should be judged by complete diagnostic pathways, including stage shift, downstream procedures, overdiagnosis, mortality, and cost effectiveness. Fourth, biological confounders such as clonal hematopoiesis and uncertain tissue of origin, together with preanalytical and cross‐platform variability, require standardized mitigation and reporting. Fifth, AI and multimodal integration should undergo external, population‐diverse, prospectively monitored validation with explicit calibration, uncertainty, and model‐governance requirements. Finally, reimbursement, health‐system capacity, equity, privacy, and postimplementation surveillance should be incorporated into the evidence framework before widespread adoption rather than treated as downstream considerations.

The future of liquid biopsy will therefore depend less on producing ever more sensitive or complex assays than on demonstrating when a circulating signal is sufficiently reliable and actionable to improve care. A clinically mature liquid‐biopsy ecosystem will integrate molecular measurement with tissue pathology, imaging, conventional diagnostics, and longitudinal clinical context while preserving clear evidence boundaries between established practice and exploratory innovation. Achieving this transition from detectability to utility is the central requirement for liquid biopsy to become a reproducible, scalable, and responsible component of precision medicine across oncology and nononcologic disease.

## Author Contributions

**Jiachen Liu**: writing – review and editing, methodology, and formal analysis. **Dianjie Zeng**: writing – original draft and conceptualization. **Keran Zhao**: writing – original draft. **Yuxi Wang**: writing – original draft. **Huijie Hu**: writing – original draft. **Fei Deng**: writing – review and editing and formal analysis. **Yinhuai Wang**: writing – review and editing and formal analysis. **Liya Sun**: writing – review and editing. **Liangmin Fu**: writing – review and editing and conceptualization. All authors have read and approved the final manuscript.

## Funding

This work was supported by the National Natural Science Foundation of China (grant no. 82503496), the Postdoctoral Fellowship Program for Innovative Talents and China Postdoctoral Science Foundation (grant no. BX2026406), the Furong Program for Young Talents in Scientific and Technological Innovation (grant no. 2026RC3048), the Hunan Provincial Natural Science Foundation (grant no. 2026jj70012), the Medical Scientific Research Foundation of Guangdong Province of China (grant no. A2025206), and the China Postdoctoral Science Foundation (grant no. 2025M782325).

## Ethics Statement

The authors have nothing to report.

## Conflicts of Interest

The authors declare no conflicts of interest.

## Data Availability

No new data were generated or analyzed in support of this research.
